# Unveiling temporal trends and disparities in mortality with co-listed coronary artery disease and cancer, 1999–2024: insights from the CDC WONDER multiple cause of death database

**DOI:** 10.1186/s40959-026-00517-8

**Published:** 2026-05-28

**Authors:** Saifullah Khan, Muhammad Hussain, Muhammad Hassan, Nisha Khalid, Javeria Nawaz, Maria Baig, FNU Pirih, Ahmad Anees Qureshi, Zona Shaikh, Sherif Eltawansy, Stephen J. Greene, Gregg C. Fonarow, Marat Fudim, Saad Ahmed Waqas, Asma Naz, Hasibullah Aminpoor

**Affiliations:** 1 Department of Dow Medical College, Dow University of Health Sciences, Karachi, Pakistan; 2 Department of Medicine, People’s University of Medical and Health Sciences, Shaheed Bennazirabad, Pakistan; 3 Division of Cardiology, Duke University School of Medicine, Durham, NC USA; 4 Duke Clinical Research Institute, Durham, NC USA; 5 Department of Medicine, Jersey Shore University Medical Center, Neptune, New Jersey USA; 6 Ahmanson-UCLA Cardiomyopathy Center, Ronald Reagan-UCLA Medical Center, Los Angeles, California USA; 7 Cardiology, Advanced Heart Failure, Transplant Duke University // Duke clinical Research, Durham, USA; 8 Faculty of Medicine, Kabul University of Medical Sciences “Abu Ali Ibn Sina”, Kabul, Afghanistan

**Keywords:** Coronary artery disease (CAD), Cancer, Mortality, Trends, Disparities

## Abstract

**Background:**

Coronary artery disease (CAD) remains one of the primary causes of morbidity and mortality worldwide. Although cancer mortality rates have declined in many high-income countries, including the United States, substantial variability persists. This study aimed to assess the trends and disparities in mortality among adults with co-listed CAD and cancer on death certificates.

**Methods:**

We conducted a retrospective analysis using the Centers for Disease Control and Prevention Wide-ranging Online Data for Epidemiologic Research (CDC WONDER) Multiple Cause-of-Death database. Data were extracted for co-listed CAD and cancer spanning from 1999 to 2024. Age adjusted mortality rates (AAMRs) were calculated. The Joinpoint Regression Program was used to evaluate annual percentage changes (APC) and average annual percentage change (AAPC) with 95% confidence intervals (CI).

**Results:**

Over 26 years, 1,321,891 deaths occurred with co-listed CAD and cancer. The overall AAMR decreased from 31.38 in 1999 to 19.77 in 2024 (AAPC: -1.88, 95% CI: -2.01 to -1.77). Men had higher mean AAMRs (36.86) than women (13.93). Among races, the highest mean AAMRs were observed in Non-Hispanic (NH) White (23.84), and NH Black (22.1), followed by NH American Indian or Alaskan Native (15.28), Hispanic (14.00), and NH Asian or Pacific Islander (11.44) individuals. Regionally, the Midwest showed higher mean AAMR (25.21), then Northeast (24.88), South (21.93), and West (21.33). Age group categorization showed highest mean AAMR in older adults (107.05), followed by middle-aged adults (6.35), and younger adults (0.18). Stratified by urbanization, rural areas exhibited higher mean AAMR (27.58) compared to urban areas (22.92). Among states, the highest mortality rates were observed in West Virginia (35.86).

**Conclusion:**

The AAMR for co-listed CAD and cancer has decreased from 1999 to 2024, with significant disparities persisting in demographics and different regions. These disparities highlight the need for targeted, equitable public health interventions to reduce mortality in these vulnerable populations.

**Graphical Abstract:**

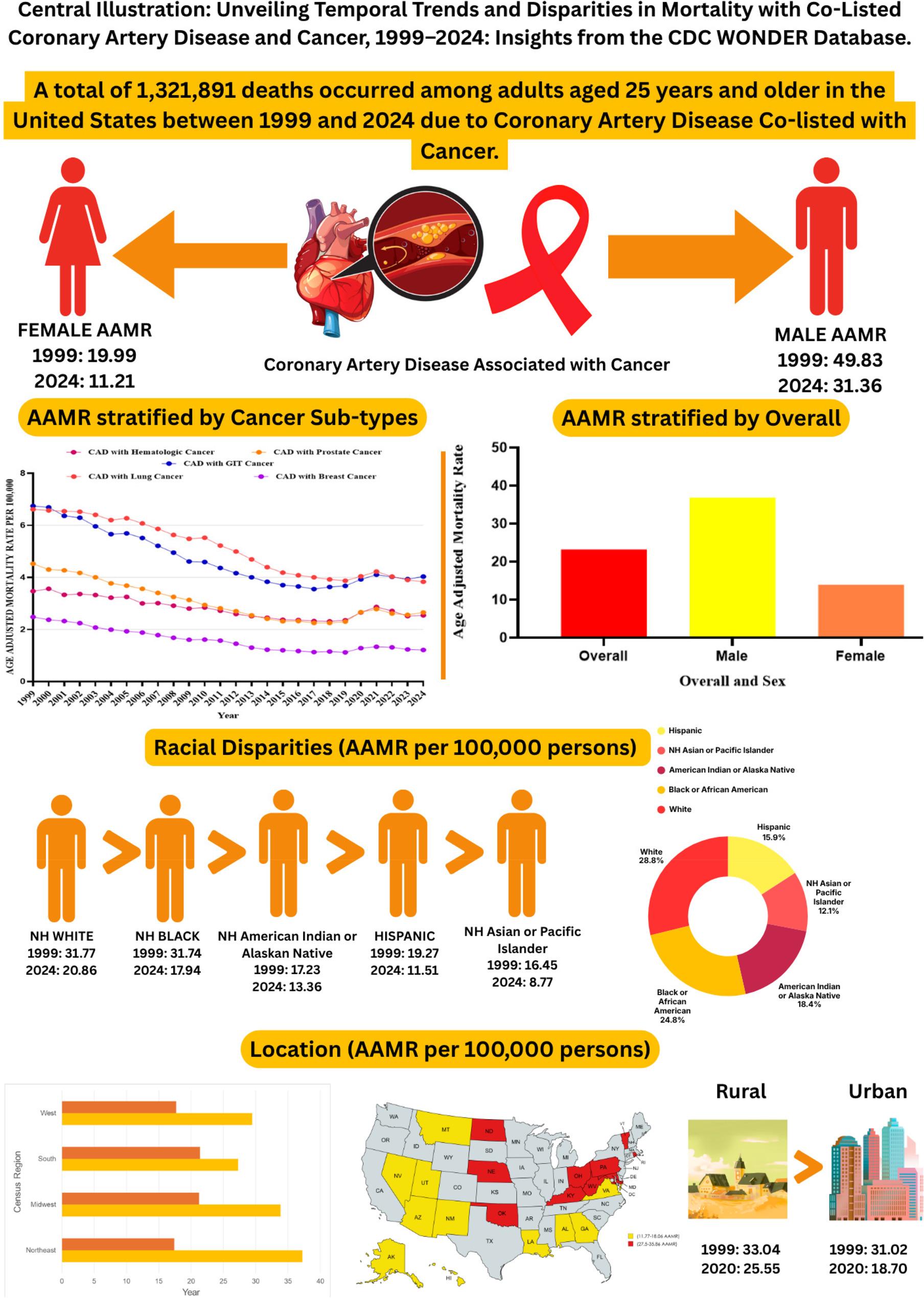

**Supplementary Information:**

The online version contains supplementary material available at 10.1186/s40959-026-00517-8.

## Introduction

CAD and its associated complications are the leading cause of death globally [1], accounting for approximately 9 million annual deaths and affecting around 126 million individuals worldwide [2]. The burden of CAD is particularly high in low- and middle-income countries, driven by socioeconomic changes, increased lifespan, and lifestyle-related risk factors [3]. Cancer is a leading global health challenge, responsible for almost one in six deaths (16.8%) worldwide recorded in 2022 [4]. Currently, low-middle income countries experience high cancer-related mortality. However, high-income countries, especially the United States, are experiencing increasing rates of cancer incidence due to population aging and lifestyle-related factors [5].

CAD and cancer frequently coexist as major contributors to global mortality. While prior literature has noted potential links between these conditions, including shared risk factors such as obesity, hypertension, diabetes, smoking, and hyperlipidemia, as well as possible contributions from certain cancer therapies, these observations primarily serve as contextual background rather than mechanistic focus in population-level analyses [6–8].

Despite extensive research on CAD and cancer individually, there remains limited population-level characterization of mortality where both conditions are co-listed on death certificates. Therefore, we conducted an analysis using the CDC WONDER database to describe temporal trends and patterns of mortality among decedents with co-listed CAD and cancer from 1999 to 2024. This study focuses on demographic and regional variations to provide insight into the evolving burden of co-occurring CAD and cancer at the population level.

## Methods

### CDC WONDER query parameters

Mortality data were obtained from the Centers for Disease Control and Prevention Wide-ranging Online Data for Epidemiologic Research (CDC WONDER) Multiple Cause-of-Death database (1999–2024 release), using the “Multiple Cause of Death, 1999–2024” dataset. Queries were structured to ensure reproducibility by applying consistent parameters across all extractions. The analysis was restricted to decedents aged ≥ 25 years during the period 1999–2024. The underlying cause of death was not restricted (i.e., all causes were included), while the multiple cause-of-death fields were used to identify records containing both coronary artery disease (CAD; ICD-10 codes I20–I25) and cancer (ICD-10 codes C00–D48), specified using a logical condition requiring the simultaneous presence of both code groups within the same death record. Data were stratified by year, sex, race/ethnicity, geographic region, urbanization status, and state by selecting each variable sequentially in the “Group By” function while maintaining identical query parameters. Output options included both death counts and age-adjusted mortality rates (AAMRs), with rates calculated per 100,000 population using the direct standardization method based on the 2000 U.S. standard population (CDC WONDER default setting). Cells with suppressed counts (death count < 10) or those flagged as unreliable were excluded in accordance with CDC data use guidelines. All results were exported as tab-delimited text files and subsequently processed for statistical analysis. To ensure consistency and reproducibility, identical query structures were applied across all subgroup analyses, with only the grouping variable modified as required.

### Study settings and population

This study utilized the Centers for Disease Control and Prevention’s Wide-ranging Online Data for Epidemiologic Research (CDC WONDER) database to examine the national mortality patterns of co-listed CAD and cancers across the United States. The CDC WONDER database retrieves the mortality data from death certificates across 50 states and the District of Columbia and offers a standardized system for large-scale epidemiological research. Prior studies on similar mortality records have also relied on ICD-10 codes for identifying trends associated with multiple causes of death [9].

For this methodology, we extracted and analyzed the statistics from the Multiple Cause-of-Death (MCD) Public Use dataset, specifically targeting records for individuals aged ≥ 25 years in which CAD ICD-10 codes (I20-I25) [10, 11] and Cancers codes (C00-D48) [12] were listed as contributing factors of death. Both CAD and cancer were identified as co-listed causes of death in the MCD and were not restricted to the underlying cause of death. The broad cancer ICD-10 code range (C00–D48) was selected to capture all cancer types listed on death certificates, including malignant neoplasms, in situ tumors, benign neoplasms, and hematologic malignancies. This approach is consistent with prior studies using the CDC WONDER Multiple Cause-of-Death dataset, allowing comprehensive identification of deaths in which cancer contributed, regardless of whether it was the underlying cause [12]. Using this full range ensures that all relevant mortality associated with co-listed CAD and cancer is included, while acknowledging that the dataset does not indicate causal relationships between conditions. The International Statistical Classification for Diseases 10th revision (ICD-10) is a systematic tool for categorizing diseases into a specific sub-category, allowing identification of both primary and contributing factors in mortality records. Moreover, this study was exempt from institutional board approval as it solely relied on deidentified and publicly available data. The study adhered to the STROBE [13] (Strengthening the Reporting of Observational Studies in Epidemiology) guidelines to ensure proper reporting of observational research.

### Data abstraction

Mortality records for individuals diagnosed with co-listed CAD and cancers were extracted for the period 1999–2024, along with demographic variables such as age, sex, and race/ethnicity. Geographic variables including state affiliations, and 2013 urbanization categories were available only for the period 1999–2020, as data beyond 2020 were excluded due to concerns regarding the reliability of age-adjusted mortality rates (AAMRs) reported after that year in the CDC WONDER MCD database. Census region data were available 1999–2024, while state and urbanization data were restricted to 1999–2020. All results were derived from this single dataset; no separate queries or differently defined datasets were used. The classifications of race/ethnicity were defined as Hispanic, Non-Hispanic (NH) White, NH Black or African American, NH Asian or Pacific Islander, and NH American Indian or Alaska Native.

Age was grouped into three categories: 25–44 years (younger adults), 45–64 years (middle-aged adults), and ≥ 65 years (older adults). To inspect variations in CAD related mortality across different cancer subtypes, we focused on five prevalent cancer subtypes: lung (C34), gastrointestinal (C15-C26), prostate (C61), breast (C50), and hematologic malignancies (C81-C96). The five cancer subtypes (lung, gastrointestinal, breast, prostate, and hematologic malignancies) were selected based on their high prevalence and contribution to overall cancer-related mortality in the United States, as well as their established relevance in cardio-oncology literature. Mortality data were further stratified based on place of death including medical facility (inpatient, outpatient or ER, dead on arrival, status unknown), decedent’s home, hospice facility, nursing home/long-term care and others (including place of death unknown).

County-level urbanization was defined according to the 2013 National Center for Health Statistics Urban-Rural Classification Scheme [14], with regions classified as either rural (micropolitan and noncore areas) or urban (large central metropolitan, large fringe metropolitan, medium metropolitan, and small metropolitan).

### Statistical analysis

The analysis was based on age-adjusted mortality rates (AAMRs) per 100,000 population, along with the annual percent change (APC) in AAMRs. Mortality rates were standardized to the 2000 U.S. population in accordance with established practices [15]. Cells with suppressed or unreliable counts (e.g., < 10 deaths) were excluded following CDC WONDER data use guidelines, and analyses were conducted on aggregated data to minimize instability in rate estimates.

Temporal trends in AAMRs were assessed using the Joinpoint Regression Program (version 5.4.0, National Cancer Institute) [16]. A grid search method was used to identify potential inflection points. The minimum number of joinpoints was set to 0 and the maximum to 4, based on the study period (1999–2024). Model selection was performed using the Weighted Bayesian Information Criterion (BIC) to determine the optimal number of joinpoints.

APCs and average annual percent changes (AAPCs) were estimated using a log-linear model, assuming a constant rate of change within each segment. Statistical significance of identified joinpoints and APC estimates was evaluated using the approximate permutation test, with a two-sided significance level of *p* < 0.05. Confidence intervals for APCs and AAPCs were calculated using the empirical quantile method based on 5001 resamples. The default heteroscedastic error structure was applied, with no adjustment for autocorrelated errors.

## Results

Between 1999 and 2024, 1,321,891 deaths occurred among adults with co-listed CAD and cancer (Supplemental Table 1). These fatalities were recorded throughout various places in the United States, with 36.9% in medical facilities, 34.4% in decedent’s home, 19.2% in nursing homes, 5.2% in hospice facilities and 4% at other places (Supplemental Table 2).

### Annual trends

**Fig. 1:**
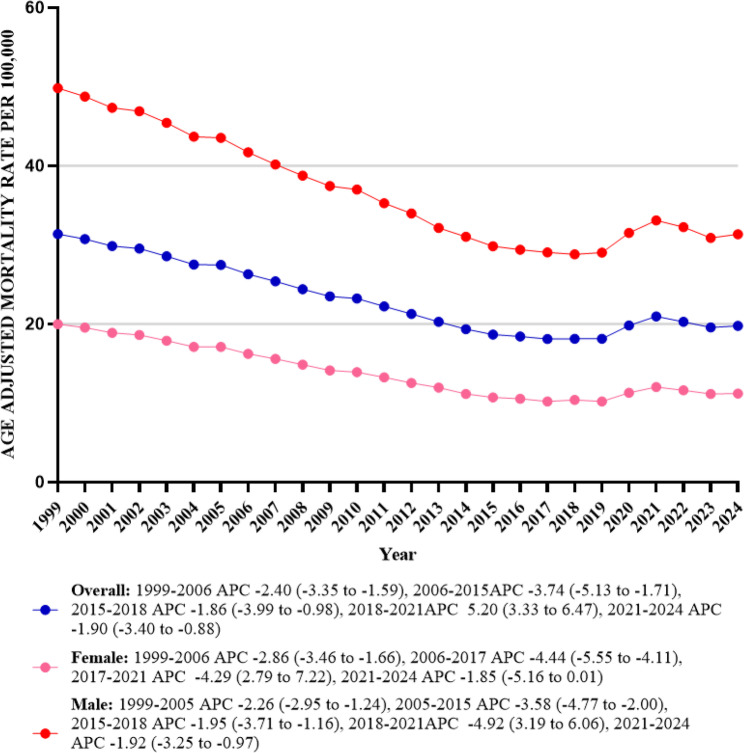
Age-adjusted mortality rate (AAMR) among adults with co-listed CAD and cancer on death certificates, stratified by overall and sex, per 100,000 population

The overall AAMR among adults with co-listed CAD and cancer on death certificates decreased from 31.38 in 1999 to 19.77 in 2024, with an AAPC of -1.88 (95% CI: -2.01 to -1.77; p value < 0.001) (Fig. 1) (Table 1). The AAMR trend declined from 1999 to 2006 (APC = -2.40; 95% CI: -3.35 to -1.59; p value = 0.003), followed by a sharper downward trend between 2006 and 2015 (APC = -3.74; 95% CI: -5.13 to -1.71; p value < 0.001), and then a trivial reduction from 2015 to 2018 (APC = -1.86; 95% CI: -3.99 to -0.98; p value = 0.009). The AAMR then increased considerably between 2018 and 2021 (APC = 5.20; 95% CI: 3.33 to 6.47; p value < 0.001) before decreasing again from 2021 to 2024 (APC = -1.90; 95% CI: -3.40 to -0.88; p value = 0.008) (Fig. 1) (Supplemental Table 3).

**Table 1:** Age-adjusted mortality rate (AAMR) and average annual percent change (AAPC) among adults with co-listed coronary artery disease and cancer on death certificates, U.S. adults aged ≥ 25 years, 1999–2024

Demographics	Deaths	Population	Mean Age-Adjusted Mortality Rate per 100,000 (95% CI)	AAPC (95% CI)
*Overall*	1,321,891	5,394,661,024	23.19 (22.99–23.39)	-1.88* (-2.01 to -1.77)
*Sex*				
Male	855,431	2,603,935,168	36.86 (36.46–37.27)	-1.88* (-2.00 to -1.77)
Female	466,460	2,790,725,856	13.93 (13.72–14.13)	-2.34* (-2.56 to -2.16)
*Race*				
NH American Indian or Alaska Native	5,814	60,683,703	15.28 (13.09–17.46)	-1.39* (-1.97 to -0.71)
NH Asian or Pacific Islander	23,521	305,418,835	11.44 (10.64–12.23)	-2.59* (-2.92 to -2.13)
NH Black	113,070	664,890,578	22.10 (21.43–22.77)	-2.49* (-2.87 to -2.13)
NH White	1,178,389	4,344,029,944	23.84 (23.62–24.06)	-1.71* (-1.84 to -1.61)
Hispanic	55,529	740,627,884	14.00 (13.37–14.64)	-2.29* (-2.55 to -2.01)
*Age Group*				
Younger adults (25–44)	3,978	2,209,338,799	0.18 (0.16–0.22)	1.35 (-0.71 to 2.94)
Middle Aged adults (45–64)	140,099	2,024,706,033	6.35 (6.18–6.52)	-1.67* (-1.87 to -1.52)
Older Adults (65+)	1,177,814	1,160,616,192	107.05 (106.06–108.04)	-1.92* (-2.04 to -1.81)
*Census Region*				
South	461,098	2,005,599,653	21.93 (21.60–22.25)	-1.07* (-1.18 to -0.95)
West	257,555	1,242,121,877	21.33 (20.91–21.75)	-2.16* (-2.32 to -2.03)
Midwest	324,049	1,158,311,489	25.21 (24.77–25.66)	-1.89* (-2.14 to -1.69)
Northeast	279,189	988,628,005	24.88 (24.41–25.35)	-3.06* (-3.21 to -2.92)
*Urbanization*				
Rural	869,558	678,634,169	27.58 (27.05–28.11)	-1.29* (-1.47 to -1.15)
Urban	230,860	3,795,213,822	22.92 (22.70–23.15)	-2.47* (-2.64 to -2.30)
*Cancer Subtypes*				
CAD-Lung Cancer	291,496	5,394,661,024	5.11 (5.02–5.21)	-2.20* (-2.36 to -2.05)
CAD-Gastrointestinal Cancer	269,129	5,394,661,024	4.72 (4.63–4.81)	-2.20* (-2.45 to -1.99)
CAD-Breast Cancer	90,662	5,394,661,024	1.60 (1.54–1.65)	-2.83* (-3.07 to -2.84)
CAD-Prostate Cancer	173,720	5,394,661,024	3.08 (3.00–3.15)	-2.32* (-2.70 to -2.03)
CAD-Hematologic Cancer	160,835	5,394,661,024	2.82 (2.74–2.89)	-1.47* (-1.67 to -1.33)

* Indicates that AAPC is statistically significant i.e. *p* value < 0.05

### Sex

The AAMR for both men and women decreased from 1999 to 2024. Among men, the AAMR decreased from 49.83 (95% CI: 49.29 to 50.38) in 1999 to 31.36 (95% CI: 31.04 to 31.68) in 2024. Similarly, among women, the AAMR decreased from 19.99 (95% CI: 19.72 to 20.25) to 11.21 (95% CI: 11.05 to 11.38).

Throughout the study timeline from 1999 to 2024, adult men exhibited higher mean AAMRs compared to adult women (mean AAMR for adult men: 36.86; 95% CI: 36.46 to 37.27; for women: 13.93; 95% CI: 13.72 to 14.13). The AAMR trend of both adult men and women decreased from 1999 to 2024 (men: AAPC: -1.88, 95% CI: -2.00 to -1.77; p value < 0.001) (women: AAPC: -2.34, 95% CI: -2.56 to -2.16; p value < 0.001).

The AAMR trend for adult women fell from 1999 to 2006 (APC = -2.86; 95% CI: -3.46 to -1.66; p value = 0.015), then a steeper reduction was observed between 2006 and 2017 (APC = -4.44; 95% CI: -5.55 to -4.11; p value < 0.001), followed by an upward shift from 2017 to 2021 (APC = 4.29; 95% CI: 2.79 to 7.22; p value = 0.013), and then a modest decline between 2021 and 2024 (APC = -1.85; 95% CI: -5.16 to 0.01; p value = 0.051). The AAMR trend for adult men decreased from 1999 to 2005 (APC = -2.26; 95% CI: -2.95 to -1.24; p value = 0.008), followed by a sharper downturn between 2005 and 2015 (APC = -3.58; 95% CI: -4.77 to -2.00; p value < 0.001), and then a marginal reduction from 2015 to 2018 (APC = -1.95; 95% CI: -3.71 to -1.16; p value = 0.005). The AAMR then showed an upward trend from 2018 to 2021 (APC = 4.92; 95% CI: 3.19 to 6.06; p value < 0.001) before shifting downward again between 2021 and 2024 (APC = -1.92; 95% CI: -3.25 to -0.97; p value = 0.008) (Fig. 1) (Supplemental Table 4).

### Race/ethnicity

The AAMR decreased across all racial/ethnic groups from 1999 to 2024: Hispanic (19.27 to 11.51); NH American Indian or Alaskan Native (17.23 to 13.36); NH Asian or Pacific Islander (16.45 to 8.77); NH White (31.77 to 20.86); and NH Black (31.74 to 17.94).

Modest disparities in AAMRs were found among different racial/ethnic groups. The highest mean AAMRs were recorded among NH White, with significant differences among the remaining racial/ethnic groups, which included NH Black, NH American Indian or Alaskan Native, Hispanic, and NH Asian or Pacific Islander (mean AAMR: NH White: 23.84, 95% CI: 23.62 to 24.06; NH Black: 22.1, 95% CI: 21.43 to 22.77; NH American Indian or Alaskan Native: 15.28, 95% CI: 13.09 to 17.46; Hispanic: 14, 95% CI: 13.37 to 14.64; NH Asian or Pacific Islander: 11.44, 95% CI: 10.64 to 12.23).

**Fig. 2:**
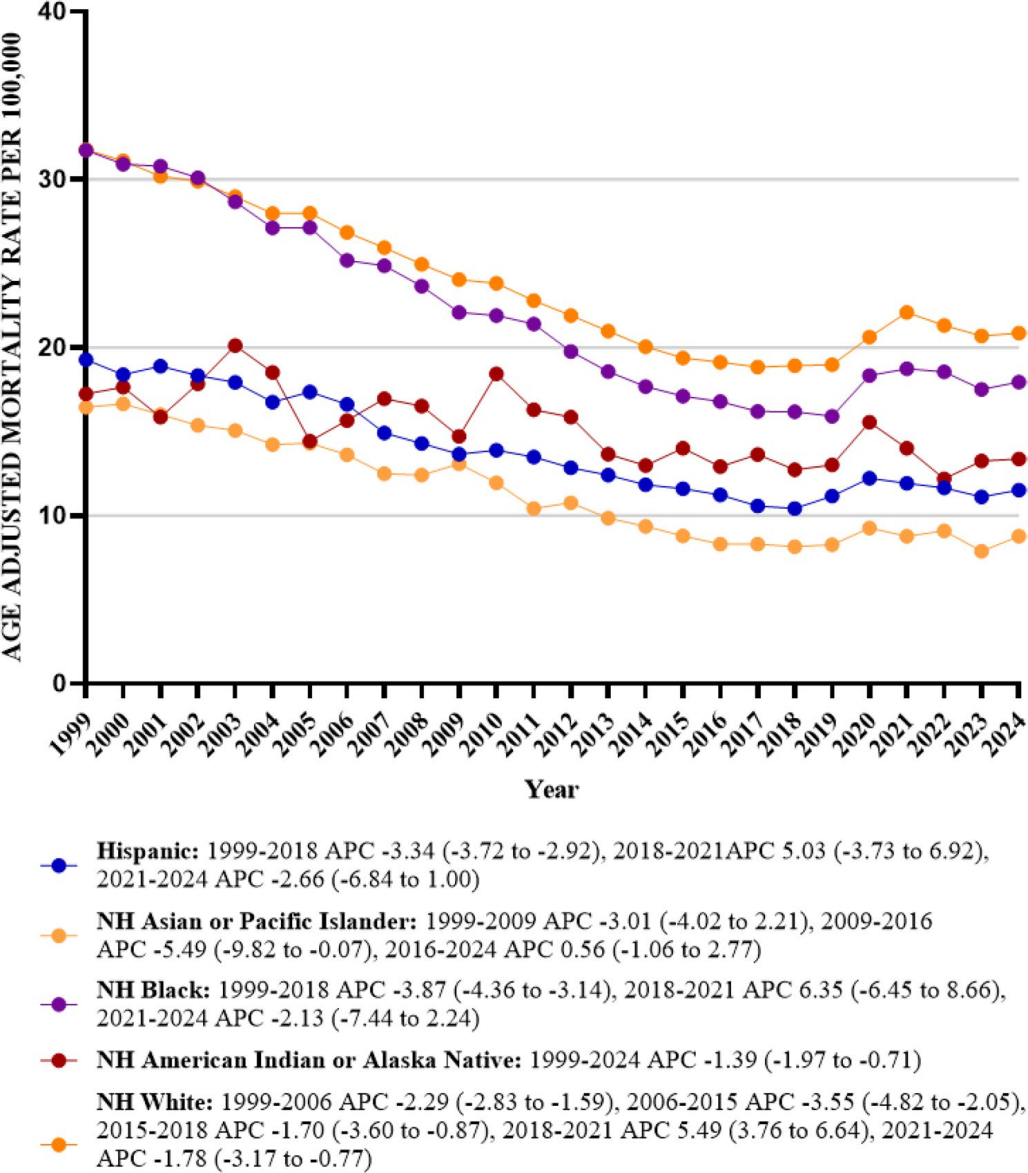
AAMR among adults with co-listed CAD and cancer on death certificates, stratified by race, per 100,000 population

Notably, the AAMR trend for all the races decreased from 1999 to 2024, with the NH Asian or Pacific Islander experiencing a greater decline than other races [NH Asian or Pacific Islander: AAPC: -2.59, (95% CI: -2.92 to -2.13; p value < 0.001); NH Black: AAPC: -2.49, (95% CI: -2.87 to -2.13; p value < 0.001); Hispanic: AAPC: -2.29, (95% CI: -2.55 to -2.01; p value < 0.001); NH White: AAPC: -1.71, (95% CI: -1.84 to -1.61; p value < 0.001); NH American Indian or Alaskan Native: AAPC: -1.39, (95% CI: -1.97 to -071; p value = 0.004). (Fig. 2) (Supplemental Table 5).

### Stratified by age

The AAMR decreased over the study period in middle-aged and older adults, while younger adults demonstrated a slight non-significant increase. Specifically, AAMR increased from 0.16 to 0.21 in younger adults, decreased from 8.14 to 5.50 in middle-aged adults, and declined from 145.86 to 91.02 in older adults. Significant differences in mean AAMRs were observed among the age groups, with older adults showing the highest mean AAMRs throughout the time period, followed by middle-aged adults and younger adults (mean AAMR: older adults: 107.05, 95% CI: 106.06 to 108.04; middle-aged adults: 6.35, 95% CI: 6.18 to 6.52; younger adults: 0.18, 95% CI: 0.16 to 0.22).

**Fig. 3:**
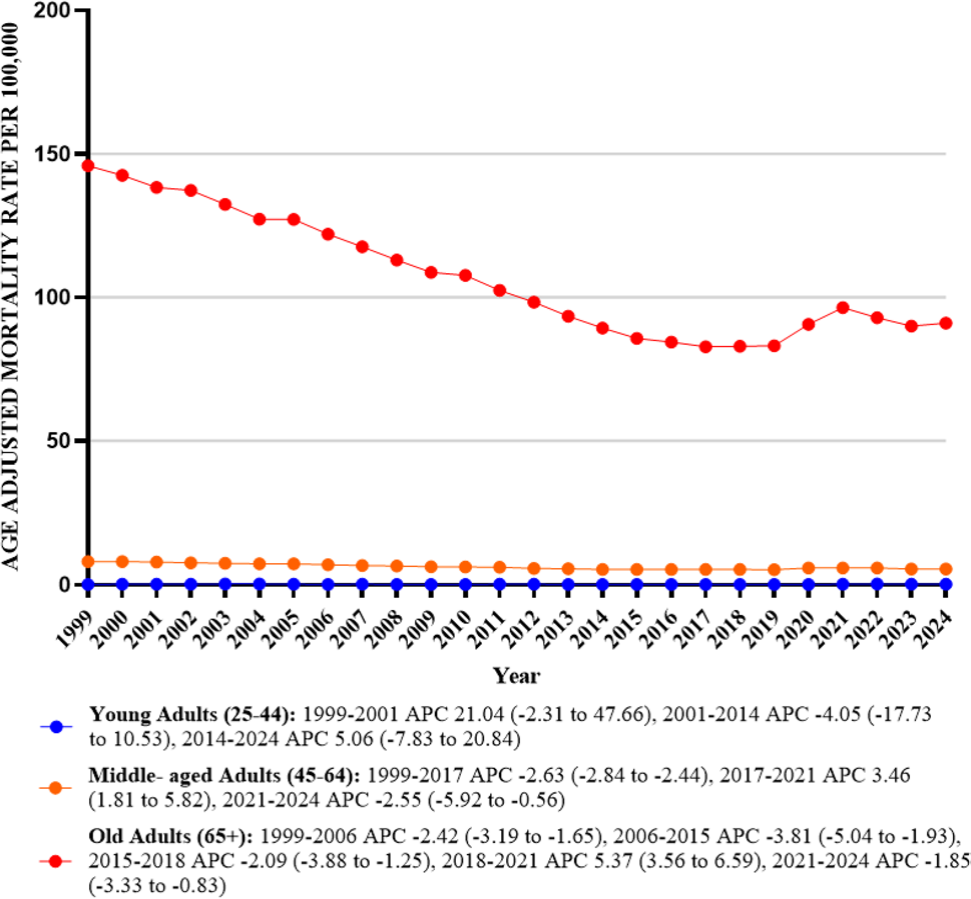
AAMR among adults with co-listed CAD and cancer on death certificates, stratified by age group, per 100,000 population

In joinpoint trend analysis, the AAMR trend for middle-aged and older adults decreased from 1999 to 2024 [(middle-aged adults: AAPC: -1.67, 95% CI: -1.87 to -1.52, *p* < 0.001; older adults: AAPC: -1.92, 95% CI: -2.04 to -1.81, *p* < 0.001)]. AAMR trend for younger adults showed a slight non-significant increase (AAPC: 1.35, 95% CI: -0.71 to 2.94, *p* = 0.103). (Fig. 3) (Supplemental Table 6).

### Geography

#### Census region

The AAMR decreased across all U.S. census regions from 1999 to 2024. In the Northeast, the AAMR dropped from 37.25 in 1999 to 17.4 in 2024; in the Midwest, from 33.87 to 21.26; in the South, from 27.26 to 21.35; and in the West, from 29.44 to 17.68.

On average, over the study period, the highest mean AAMRs were observed in the Midwest (mean AAMR: 25.21; 95% CI: 24.77 to 25.66), followed by the Northeast (mean AAMR: 24.88; 95% CI: 24.42 to 25.35), South (mean AAMR: 21.93; 95% CI: 21.6 to 22.25), and West (mean AAMR:21.33; 95% CI: 20.91 to 21.75).

**Fig. 4:**
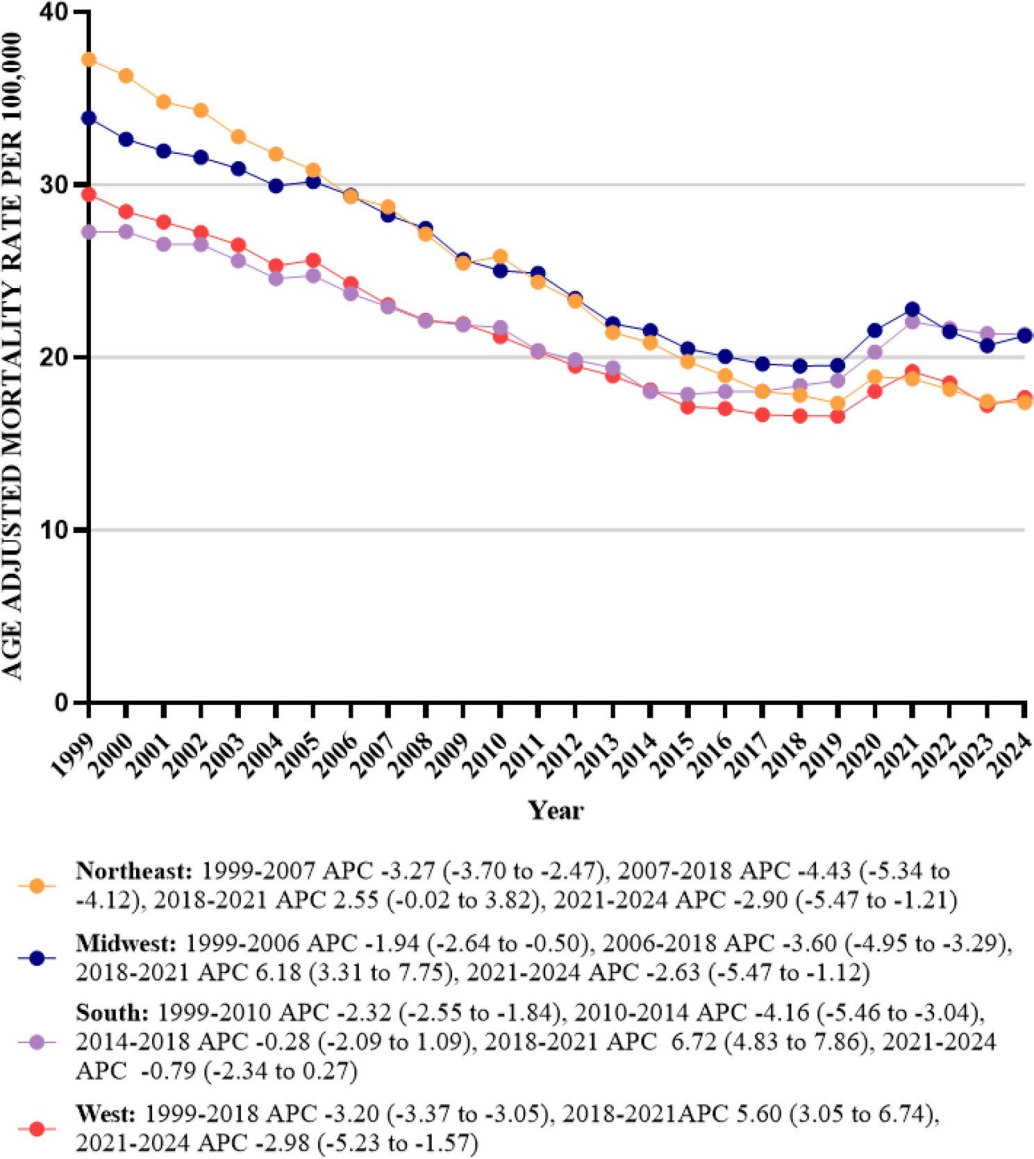
AAMR among adults with co-listed CAD and cancer on death certificates, stratified by census region, per 100,000 population

The AAMR of all four regions showed a decrease from 1999 to 2024, with the decrease most pronounced in the Northeast region [Northeast: AAPC: -3.06, (95% CI: -3.21 to -2.92; p value < 0.001); West: AAPC:-2.16, (95% CI: -2.32 to -2.03; p value < 0.001); Midwest: AAPC: -1.89, (95% CI: -2.14 to -1.69; p value < 0.001); South: AAPC: -1.07, (95% CI: -1.18 to -0.95; p value < 0.001)]. (Fig. 4) (Supplemental Table 7).

#### Urbanization

The AAMR decreased in both urban and rural areas from 1999 to 2020. In urban areas, the AAMR decreased from 31.02 in 1999 to 18.7 in 2020, while in rural areas it decreased from 33.04 to 25.55.

**Fig. 5:**
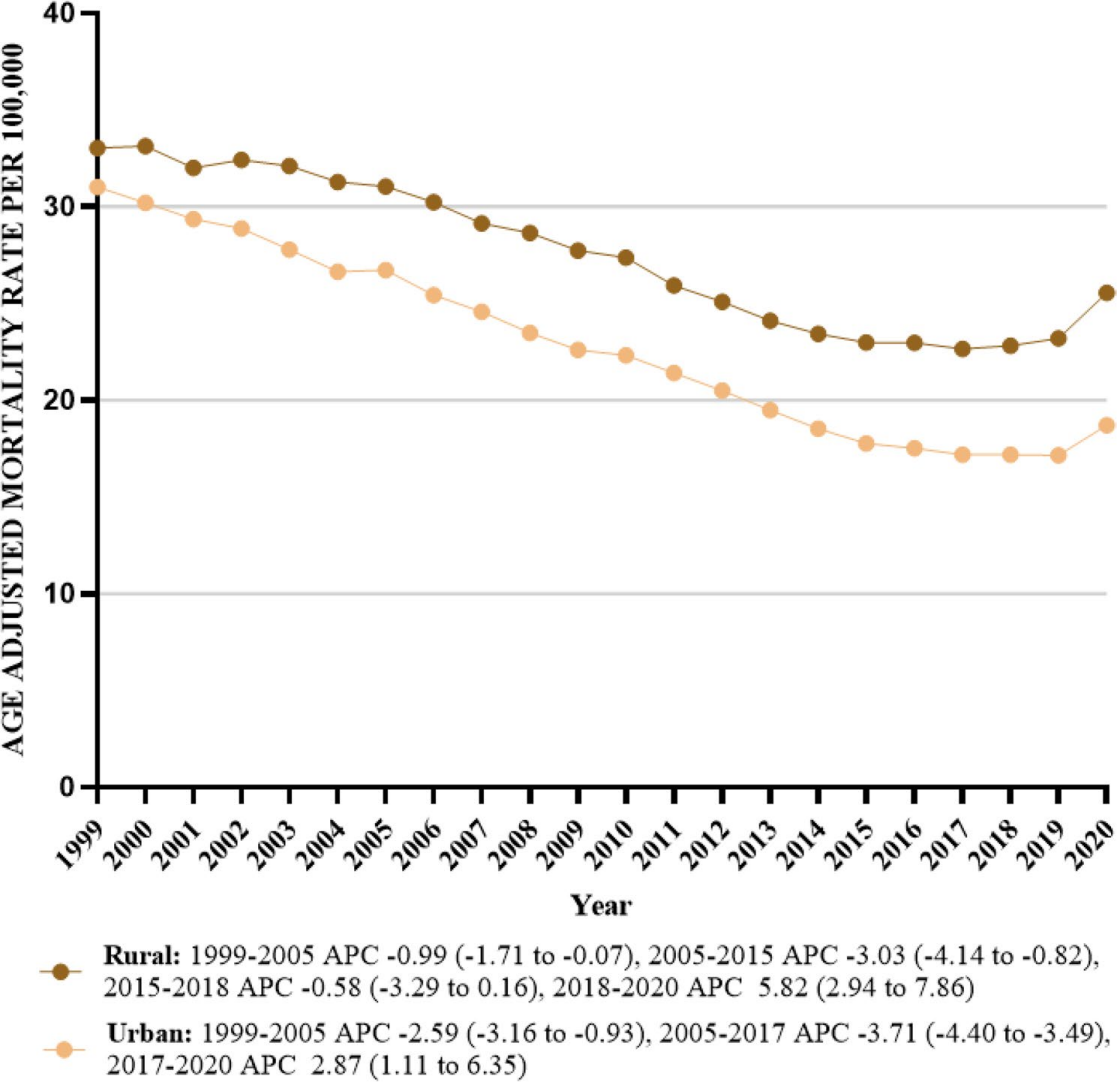
AAMR among adults with co-listed CAD and cancer on death certificates, stratified by urbanization status, per 100,000 population

Rural areas showed higher AAMRs throughout the study timeline, with a mean AAMR of 27.58 for rural (95% CI: 27.05 to 28.11) and 22.92 for urban (95% 22.7 to 23.15). The AAMRs of both rural and urban areas decreased from 1999 to 2020 with urban areas showing a more pronounced decline than rural areas. [(urban: AAPC: -2.47, 95% CI: -2.64 to -2.30; p value < 0.001) (rural: AAPC: -1.29, 95% CI: -1.47 to -1.15; p value < 0.001)]. (Fig. 5) (Supplemental Table 8).

#### State

**Fig. 6:**
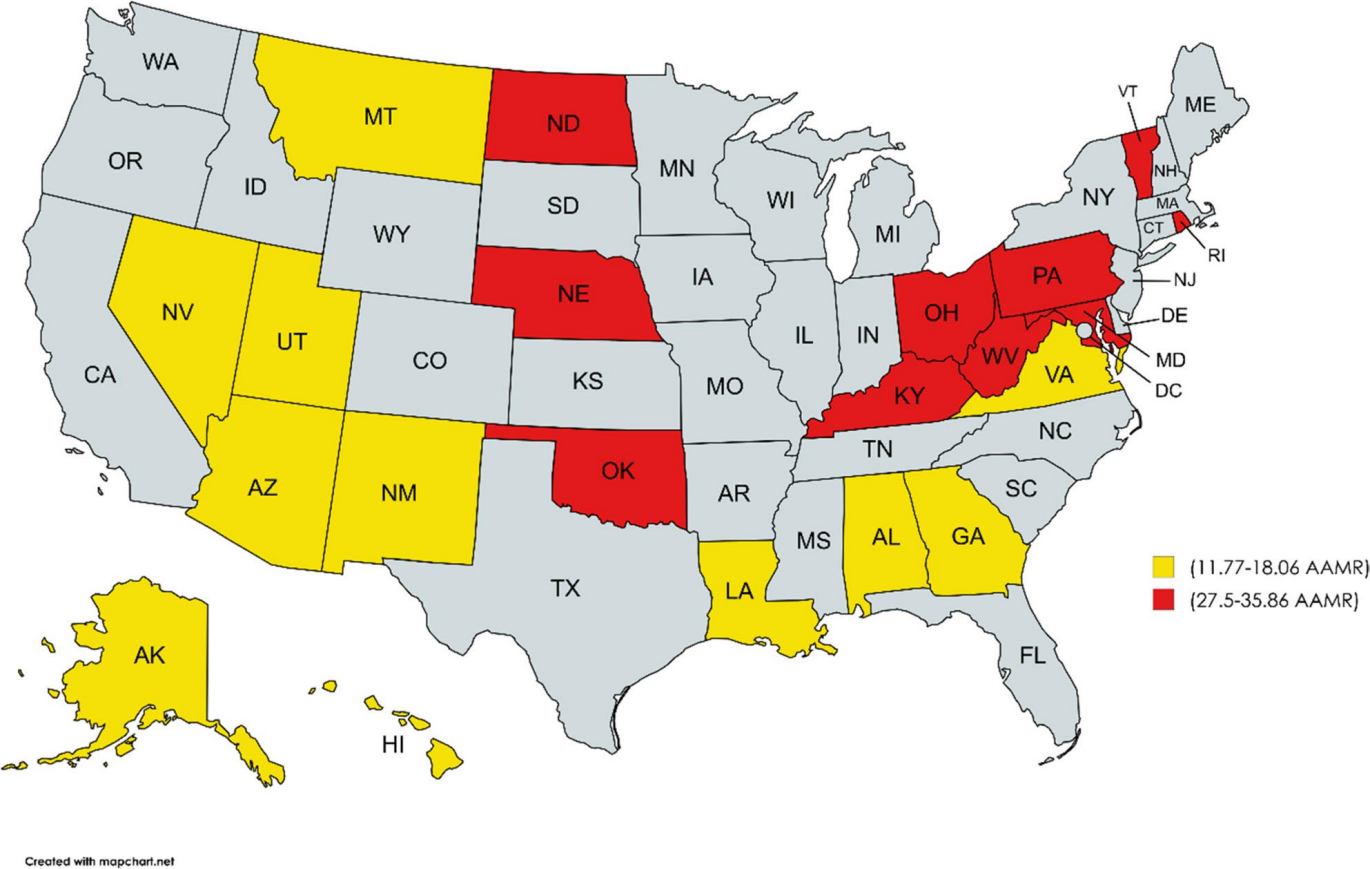
AAMR among adults with co-listed CAD and cancer on death certificates, stratified by state, per 100,000 population

State-level variation in AAMRs was assessed for the period 1999–2020. Disparities in AAMRs were noticed among different states with AAMRs ranging from as low as 11.77 (95% CI: 11.37 to 12.17) in Utah to as high as 35.86 (95% CI: 35.22 to 36.5) in West Virginia. States falling within the top 90th percentile included North Dakota, Ohio, Oklahoma, Rhode Island and Vermont which had approximately three times higher AAMRs compared to States in the lower 10th percentile which included Arizona, Georgia, Hawaii, Louisiana and Nevada (Fig. 6) (Supplemental Table 9).

### Cancer subtypes

Overall, the highest AAMRs regarding different types of cancer were observed with lung cancer (AAMR: 5.11; 95% CI: 5.02 to 5.21), followed by gastrointestinal cancer (AAMR: 4.72; 95% CI: 4.63 to 4.81), prostate cancer (AAMR: 3.08; 95% CI: 3 to 3.15) and hematological cancers (AAMR: 2.82; 95% CI: 2.74 to 2.89). The lowest AAMR was observed in breast cancer (AAMR: 1.6, 95% CI: 1.54 to 1.65).

**Fig. 7:**
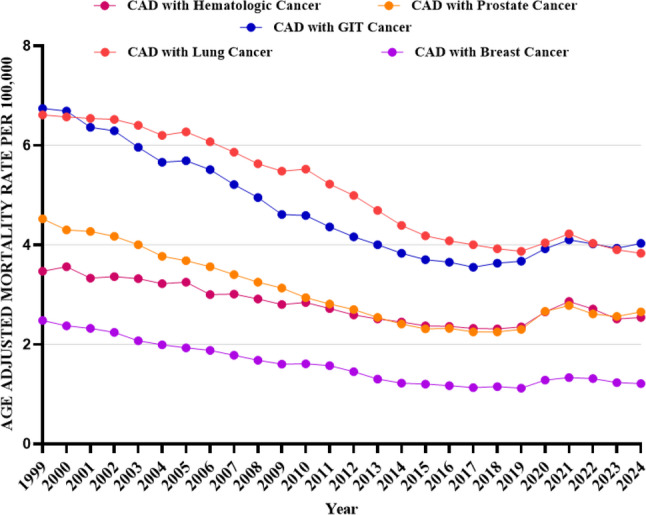
AAMR among adults with co-listed CAD and cancer on death certificates, stratified by cancer subtype, per 100,000 population

AAMRs for all cancer subtypes co-listed with coronary artery disease on death certificates decreased overall from 1999 to 2024. The greatest decline was seen in breast cancer (AAPC: -2.83; 95% CI: -3.07 to -2.64; p value < 0.001), followed by prostate cancer (AAPC: -2.32; 95% CI: -2.70 to -2.03; p value < 0.001). Similar decreases were seen in gastrointestinal cancer (AAPC: -2.20; 95% CI: -2.45 to -1.99; p value < 0.001) and lung cancer (AAPC: -2.20; 95% CI: -2.36 to -2.05; p value < 0.001), followed by hematological cancer (AAPC: -1.47; 95% CI: -1.67 to -1.33; p value < 0.001) (Fig. 7) (Supplemental Table 10).

Overall, co-listed CAD and cancer mortality demonstrated a significant declining trend from 1999 to 2024. However, notable disparities persisted across sex, race/ethnicity, age groups, and geographic regions, with the highest burden observed among men, older adults, and rural populations. A transient increase in mortality between 2018 and 2021 highlights potential healthcare disruptions during this period.

## Discussion

This nationwide CDC WONDER analysis highlights critical trends and disparities in AAMR for CAD and co-listed cancer mortality among U.S. adults aged ≥ 25. The overall AAMR for co-listed CAD and cancer has declined from 1999 to 2024, but with a significant upward trend observed between 2018 and 2021. Significant differences in the mortality rates were observed between sexes, with men having higher overall mortality rates. Additionally, important racial disparities were discovered during the analysis, with NH Black and NH White individuals having the highest mortality rates, followed by Hispanics, American Indians or Alaska Natives, and NH Asian or Pacific Islander individuals. Moreover, older adults showed almost 17 times higher mortality rates than middle-aged individuals. The burden of mortality remains disproportionately higher among individuals from the Midwest and West Virginia (Graphical Abstract). States falling in the top 90th percentile include North Dakota, Ohio, Oklahoma, Rhode Island, and Vermont. In comparison, those in the lower 10th percentile include Arizona, Georgia, Hawaii, Louisiana, and Nevada. Furthermore, rural areas showed consistently higher mortality rates compared to urban populations. These findings may help inform targeted measures and public health strategies to reduce the burden of mortality among vulnerable populations. Importantly, as a multiple cause-of-death analysis, this study is descriptive in nature and cannot establish causal relationships, temporality between CAD and cancer, or underlying biological mechanisms.

While prior literature suggests a potential bidirectional relationship between CAD and cancer, the present analysis cannot establish such relationships and instead describes temporal trends in co-listed mortality. Evidence has suggested that patients with CAD have been reported to have higher observed incidence of developing cancer due to shared risk factors such as obesity, smoking, sedentary lifestyle, and systemic inflammation, which promotes both atherosclerosis and tumorigenesis [17–19]. A systematic review and meta-analysis of cohort studies found that individuals with CAD have higher odds of developing cancers compared to those without CAD [20]. Similarly, a study observed that post-discharge cancer significantly increases cardiovascular mortality in CAD patients, particularly lung cancer, which is associated with nearly a twofold increase in cardiovascular death [21]. The annual trend in mortality for CAD among cancer patients has declined over the past two decades, reflecting advances in cardiovascular and oncologic care [22, 23], including early detection and optimized therapies. However, a temporary rise was observed during COVID-19. This period of excess mortality may likely be attributed to disruptions in routine healthcare, delayed cancer diagnosis and intervention, reduced access to specialty care, and increased vulnerability among patients with pre-existing cardiovascular conditions [24–26].

Between 1999 and 2024, AAMR for co-listed CAD and cancer declined for both men and women. Throughout the study period, men consistently showed higher mortality rates than women. The complex interplay between biological, social, and behavioral factors may explain the higher rate of mortality observed among men. The observed sex-based differences in mortality may reflect a combination of behavioral, clinical, and healthcare access factors reported in prior studies; however, these mechanisms cannot be directly assessed using the present dataset. Life factors, including the prevalence of smoking, alcohol consumption, and environmental exposure to carcinogens, are relatively higher among men than women, resulting in a higher prevalence of cancer among men and subsequent death [27, 28]. Moreover, men generally have a higher prevalence of cardiovascular risk factors and CAD, which likely contributes to the higher mortality [29]. Notably, both sexes experienced a transient increase in AAMR between 2017 and 2021, coinciding with the COVID-19 pandemic, as discussed previously [30]. Specific preventive measures and broader public health strategies are needed to reduce the mortality burden, especially among adult men.

Throughout the study period, important racial and ethnic disparities were observed, with NH White and NH Black individuals having the highest mortality rates. The observed racial and ethnic disparities likely reflect a complex interplay of socioeconomic, healthcare access, and clinical factors described in prior literature; however, the current analysis cannot determine the underlying causes of these differences [31–33]. In contrast, NH White individuals, having similar higher mortality rates as NH Black individuals, can attribute this to their lifestyle factors, including increased prevalence of smoking, alcohol consumption, and a higher elderly population [34–36]. These findings may help inform targeted public health awareness campaigns and increased access to healthcare among NH Black individuals, which may help reduce mortality in these populations.

When stratified by age groups, the higher mortality observed among older adults likely reflects greater comorbidity burden and overall vulnerability reported in prior studies, although such contributing factors cannot be directly evaluated in this analysis [37–39]. Middle-aged adults exhibited intermediate risk, while younger adults were least affected. Furthermore, age-related endothelial dysfunction, systemic inflammation, and reduced cardiac reserve further compound the risk of mortality [40, 41]. Despite national improvement since 1999, substantial inequalities in mortality from co-listed CAD and cancer persist across U.S. states, regions, and urbanization levels. Certain states, such as West Virginia, carry the heaviest burden, consistent with prior evidence indicating West Virginia as among the states with the highest cancer incidence [42], while others remain comparatively at lower rates.

Regional analysis reveals that the Midwest and Northeast experienced higher mortality burdens compared to the South and West. Although all regions have shown improvement since 1999, the pace and trajectory of improvement have differed, reflecting heterogeneity in socioeconomic conditions and healthcare access [43]. Similar geographical differences have been reported for both cardiovascular disease and cancer individually [42, 44]. Urban and rural disparities were also striking. Rural residents face persistently higher mortality from co-listed CAD and cancer than urban residents, with slower gains in recent years. Geographic and urban–rural disparities observed in this study are consistent with prior reports, though the present dataset does not allow evaluation of the specific factors contributing to these differences [45, 46].

Differences across cancer subtypes may reflect variation in disease severity and clinical characteristics reported in prior studies; however, mechanistic explanations cannot be determined from this dataset [47]. Lung, gastrointestinal, and hematological cancers exhibit the highest mortality rates, while breast cancer is less detrimental. These findings align with prior studies on co-listed CAD and cancer, indicating that advanced-stage malignancies and cancer subtypes are associated with increased cardiovascular mortality [48].

### Future directions

This study is descriptive and does not establish causality; thus, clinical recommendations cannot be derived directly. However, the co-occurrence of CAD and cancer mortality highlights the need for further research into shared risk factors such as hypertension, dyslipidemia, and obesity [49]. Existing evidence suggests that in patients receiving cardiotoxic cancer therapies, closer cardiac monitoring and cardioprotective agents (ACE inhibitors, ARBs, beta-blockers) may be considered in high-risk settings [50]. Multidisciplinary cardio-oncology programs have also been proposed to enhance coordinated care and surveillance [51]. These should be viewed as general context from prior literature rather than implications of the present analysis.

### Limitations

While the present study provides valuable insights, it is not without its limitations. The dependency on the CDC WONDER database induces potential reporting biases and the risk of misclassifying cause-specific mortality, especially in individuals with several comorbid conditions. The study’s reliance on aggregated nationwide data further limits its ability to regional and local inconsistencies in healthcare access, diagnostic methodology, and treatment interventions, leading to a lack of representation of marginalized populations. Additionally, this study cannot establish causal relationships or underlying biological mechanisms, and all interpretations beyond descriptive trends should be considered hypothesis-generating. The absence of in-depth clinical details, such as cancer stage or care protocols, further limits the study’s ability to analyze the role these factors play in impacting mortality outcomes. Moreover, innovations in medical care—particularly in cancer and cardiovascular care—over the study’s timeframe could be inadequately captured, possibly affecting observed trends. Uncaptured confounding factors, such as socioeconomic status and health-related behaviors, might further shape the mortality outcomes. The lack of urbanization data from 2020 onwards restricts our ability to perform trend analysis throughout study period. Lastly, the racial and ethnic classification used in the dataset might not adequately capture demographic diversity, and the impact of the COVID-19 pandemic may have resulted in distorted trends, given its disruption of healthcare provision and diagnostic delays.

## Conclusion

Our study revealed a substantial decline in AAMR among adults with co-listed CAD and cancer from 1999 to 2024. However, this progress was interrupted by a significant but temporary increase in mortality rates from 2018 to 2021. These trends underscore persistent health disparities, with disproportionately higher burden observed among male, older adults, NH White and Black populations, and individuals residing in rural areas and the Midwest. This complex mortality landscape calls for a multifactorial public health strategy that strengthens integrated cardio-oncology care to implement more resilient and equitable healthcare policies. These findings emphasize the urgent need for targeted, equity-focused cardio-oncology strategies to address persistent disparities.

## Supplementary Information

Supplementary Material 1.

## Data Availability

The data supporting the findings of this study were obtained from the CDC WONDER online database (Centers for Disease Control and Prevention Wide-ranging Online Data for Epidemiologic Research). The datasets used and analyzed during the current study are publicly available and can be accessed at [CDC WONDER] (https://wonder.cdc.gov).
