# Supplementary material for: Unveiling temporal trends and disparities in mortality with co-listed coronary artery disease and cancer, 1999–2024: insights from the CDC WONDER multiple cause of death database
Source: Cardiooncology. 2026 May 28;12:100. doi: 10.1186/s40959-026-00517-8 (PMC13411586; doi:10.1186/s40959-026-00517-8)
Supplement: Supplementary file 1 — Supplementary Material 1. [file 40959_2026_517_MOESM1_ESM.docx]

| **Supplemental Table 1. Coronary Artery Disease–related mortalities identified via multiple cause of death (MCOD) coding in adults with cancer, stratified by sex and race, in the United States, 1999–2024.** | | | | | | | | | |  |
| --- | --- | --- | --- | --- | --- | --- | --- | --- | --- | --- |
| **Supplemental Table 1. Coronary Artery Disease–related mortalities identified via multiple cause of death (MCOD) coding in adults with cancer, stratified by sex and race, in the United States, 1999–2024.** | | | | | | | | | | |
| **Deaths** | | | | | | | | | | |
| **Year** | **Overall** | **Women** | **Men** | **NH American Indian or Alaska Native** | **NH Asian or Pacific Islander** | **NH Whites** | **Hispanic** | **NH Blacks** | **Population** | |
| **1999** | 55378 | 21820 | 33558 | 124 | 602 | 50060 | 1474 | 4592 | 180408769 | |
| **2000** | 54861 | 21547 | 33314 | 149 | 639 | 49507 | 1491 | 4566 | 181984640 | |
| **2001** | 54097 | 21066 | 33031 | 136 | 667 | 48665 | 1629 | 4629 | 184305128 | |
| **2002** | 54276 | 20947 | 33329 | 159 | 703 | 48848 | 1673 | 4566 | 186208028 | |
| **2003** | 53328 | 20348 | 32980 | 182 | 725 | 47953 | 1724 | 4468 | 188090429 | |
| **2004** | 52078 | 19670 | 32408 | 181 | 722 | 46872 | 1735 | 4303 | 190205384 | |
| **2005** | 52987 | 19937 | 33050 | 150 | 793 | 47635 | 1870 | 4409 | 192551384 | |
| **2006** | 51661 | 19232 | 32429 | 177 | 801 | 46484 | 1870 | 4199 | 195019359 | |
| **2007** | 50792 | 18768 | 32024 | 187 | 766 | 45572 | 1770 | 4267 | 197403777 | |
| **2008** | 49837 | 18149 | 31688 | 198 | 806 | 44655 | 1816 | 4178 | 199795090 | |
| **2009** | 48928 | 17558 | 31370 | 187 | 904 | 43798 | 1814 | 4039 | 202107016 | |
| **2010** | 49167 | 17542 | 31625 | 238 | 859 | 44002 | 1908 | 4068 | 203891983 | |
| **2011** | 48310 | 17064 | 31246 | 232 | 824 | 43101 | 2018 | 4153 | 206592936 | |
| **2012** | 47529 | 16497 | 31032 | 235 | 888 | 42408 | 2030 | 3998 | 208826037 | |
| **2013** | 46479 | 16077 | 30402 | 212 | 884 | 41492 | 2088 | 3891 | 211085314 | |
| **2014** | 45567 | 15314 | 30253 | 217 | 902 | 40583 | 2134 | 3865 | 213809280 | |
| **2015** | 44956 | 14979 | 29977 | 243 | 920 | 39913 | 2196 | 3880 | 216553817 | |
| **2016** | 45390 | 15055 | 30335 | 234 | 916 | 40301 | 2246 | 3939 | 218641417 | |
| **2017** | 45778 | 14855 | 30923 | 267 | 977 | 40543 | 2242 | 3991 | 221447331 | |
| **2018** | 47004 | 15428 | 31576 | 261 | 997 | 41686 | 2298 | 4060 | 223311190 | |
| **2019** | 48251 | 15541 | 32710 | 285 | 1076 | 42718 | 2564 | 4172 | 224981167 | |
| **2020** | 53764 | 17414 | 36350 | 350 | 1278 | 47205 | 2948 | 4931 | 226635013 | |
| **2021** | 55165 | 17930 | 37235 | 299 | 1145 | 48557 | 2936 | 4912 | 228238412 | |
| **2022** | 56222 | 18370 | 37852 | 281 | 1281 | 49315 | 3002 | 5073 | 229508599 | |
| **2023** | 54805 | 17663 | 37142 | 316 | 1164 | 48119 | 2983 | 4918 | 231529762 | |
| **2024** | 55281 | 17689 | 37592 | 314 | 1282 | 48397 | 3070 | 5003 | 231529762 | |
| **Total** | **1321891** | **466460** | **855431** | **5814** | **23521** | **1178389** | **55529** | **113070** | **5394661024** | |

**Supplemental Table 2.** Coronary Artery Disease–related mortalities based on MCOD data, stratified by place of death, in adults with cancer in the United States, 1999–2024.

| **Supplemental Table 2. Coronary Artery Disease–related mortalities based on MCOD data, stratified by place of death, in adults with cancer in the United States, 1999–2024.** | | | | | |
| --- | --- | --- | --- | --- | --- |
| **Deaths** | | | | | |
| **Year** | **Medical Facility** | **Nursing Home/Long-term Care Facility** | **Hospice Facility** | **Decedent's Home** | **Others** |
| **1999** | 26289 | 12608 | - | 14739 | 1727 |
| **2000** | 25799 | 12606 | - | 14706 | 1743 |
| **2001** | 25073 | 12258 | - | 14735 | 2016 |
| **2002** | 24470 | 12387 | - | 15225 | 2185 |
| **2003** | 23558 | 12007 | 144 | 15274 | 2194 |
| **2004** | 22241 | 11697 | 259 | 15280 | 2452 |
| **2005** | 22277 | 11874 | 793 | 15755 | 2165 |
| **2006** | 21295 | 11570 | 1144 | 15474 | 2019 |
| **2007** | 20642 | 11057 | 1613 | 15473 | 1937 |
| **2008** | 19593 | 10697 | 1837 | 15170 | 2029 |
| **2009** | 18259 | 10381 | 1992 | 15418 | 2129 |
| **2010** | 18409 | 10193 | 2415 | 15821 | 2294 |
| **2011** | 17713 | 9857 | 2668 | 15862 | 2181 |
| **2012** | 16680 | 9238 | 3245 | 16104 | 2232 |
| **2013** | 15755 | 8937 | 3155 | 16350 | 2247 |
| **2014** | 14984 | 8792 | 3377 | 16604 | 1776 |
| **2015** | 14873 | 8360 | 3778 | 16375 | 1553 |
| **2016** | 14857 | 8116 | 3989 | 16802 | 1624 |
| **2017** | 14460 | 8104 | 4178 | 17298 | 1721 |
| **2018** | 14598 | 8199 | 4238 | 18141 | 1814 |
| **2019** | 14789 | 8017 | 4576 | 18914 | 1950 |
| **2020** | 15473 | 7435 | 4414 | 24114 | 2317 |
| **2021** | 16548 | 7068 | 4787 | 24452 | 2298 |
| **2022** | 17103 | 7424 | 5062 | 24137 | 2489 |
| **2023** | 16279 | 7615 | 5334 | 23328 | 2247 |
| **2024** | 16385 | 7693 | 5410 | 23544 | 2242 |
| **Total** | **488402** | **254190** | **68408** | **455095** | **53581** |

**Supplemental Table 3. Annual percent change (APC) of coronary artery disease–related age-adjusted mortality rates per 100,000, derived from MCOD, in adults with cancer in the United States, 1999–2024.**

| **Supplemental Table 3. Annual percent change (APC) of coronary artery disease–related age-adjusted mortality rates per 100,000, derived from MCOD, in adults with cancer in the United States, 1999–2024.** | | |
| --- | --- | --- |
| **Year Interval** | **APC (95% CI)** | **P-value** |
| **Overall** | | |
| 1999-2006 | -2.40* (-3.35 to -1.59) | 0.003 |
| 2006-2015 | -3.74* (-5.13 to -1.71) | <0.001 |
| 2015-2018 | -1.86* (-3.99 to -0.98) | 0.009 |
| 2018-2021 | 5.20* (3.33 to 6.47) | <0.001 |
| 2021-2024 | -1.90* (-3.40 to -0.88) | 0.001 |
| **Men** | | |
| 1999-2005 | -2.26* (-2.95 to -1.24) | 0.008 |
| 2005-2015 | -3.58* (-4.77 to -2.00) | <0.001 |
| 2015-2018 | -1.95* (-3.71 to -1.16) | 0.005 |
| 2018-2021 | 4.92* (3.19 to 6.06) | <0.001 |
| 2021-2024 | -1.92* (-3.25 to -0.97) | 0.001 |
| **Women** | | |
| 1999-2006 | -2.86* (-3.46 to -1.66) | 0.015 |
| 2006-2017 | -4.44* (-5.55 to -4.11) | 0.0004 |
| 2017-2021 | 4.29* (2.79 to 7.22) | 0.013 |
| 2021-2024 | -1.85 (-5.16 to 0.01) | 0.051 |
| **NH White** | | |
| 1999-2006 | -2.29* (-2.83 to -1.59) | 0.003 |
| 2006-2015 | -3.55* (-4.82 to -2.05) | <0.001 |
| 2015-2018 | -1.70* (-3.60 to -0.87) | 0.006 |
| 2018-2021 | 5.49* (3.76 to 6.64) | <0.001 |
| 2021-2024 | -1.78* (-3.17 to -0.77) | 0.001 |
| **NH Black** | | |
| 1999-2018 | -3.87* (-4.36 to -3.14) | 0.03 |
| 2018-2021 | 6.35 (-6.45 to 8.66) | 0.181 |
| 2021-2024 | -2.13 (-7.44 to 2.24) | 0.201 |
| **NH American Indian or Alaska Native** | | |
| 1999-2024 | -1.39* (-1.97 to -0.71) | 0.0004 |
| **NH Asian or Pacific Islander** | | |
| 1999-2009 | -3.01 (-4.02 to 2.21) | 0.079 |
| 2009-2016 | -5.49* (-9.82 to -0.07) | 0.049 |
| 2016-2024 | 0.56 (-1.06 to 2.77) | 0.351 |
| **Hispanic** | | |
| 1999-2018 | -3.34* (-3.72 to -2.92) | 0.016 |
| 2018-2021 | 5.03 (-3.73 to 6.92) | 0.112 |
| 2021-2024 | -2.66 (-6.84 to 1.00) | 0.11 |
| **Rural areas** | | |
| 1999-2005 | -0.99* (-1.71 to -0.07) | 0.045 |
| 2005-2015 | -3.03* (-4.14 to -0.82) | 0.02 |
| 2015-2018 | -0.58 (-3.29 to 0.16) | 0.104 |
| 2018-2020 | 5.82* (2.94 to 7.86) | <0.001 |
| **Urban areas** | | |
| 1999-2005 | -2.59* (-3.16 to -0.93) | 0.015 |
| 2005-2017 | -3.71* (-4.40 to -3.49) | <0.001 |
| 2017-2020 | 2.87* (1.11 to 6.35) | 0.002 |
| **Northeast region** | | |
| 1999-2007 | -3.27* (-3.70 to -2.47) | 0.002 |
| 2007-2018 | -4.43* (-5.34 to -4.12) | <0.001 |
| 2018-2021 | 2.55 (-0.02 to 3.82) | 0.051 |
| 2021-2024 | -2.90* (-5.47 to -1.21) | 0.005 |
| **South region** | | |
| 1999-2010 | -2.32* (-2.55 to -1.84) | 0.005 |
| 2010-2014 | -4.16* (-5.46 to -3.04) | <0.001 |
| 2014-2018 | -0.28 (-2.09 to 1.09) | 0.607 |
| 2018-2021 | 6.72* (4.83 to 7.86) | <0.001 |
| 2021-2024 | -0.79 (-2.34 to 0.27) | 0.138 |
| **Midwest region** | | |
| 1999-2006 | -1.94* (-2.64 to -0.50) | 0.034 |
| 2006-2018 | -3.60* (-4.95 to -3.29) | 0.001 |
| 2018-2021 | 6.18* (3.31 to 7.75) | 0.009 |
| 2021-2024 | -2.63* (-5.47 to -1.12) | 0.013 |
| **West region** | | |
| 1999-2018 | -3.20* (-3.37 to -3.05) | <0.001 |
| 2018-2021 | 5.60* (3.05 to 6.74) | <0.001 |
| 2021-2024 | -2.98* (-5.23 to -1.57) | <0.001 |
| **Younger Adults (25-44)** | | |
| 1999-2001 | 21.04 (-2.31 to 47.66) | 0.165 |
| 2001-2014 | -4.05 (-17.73 to 10.53) | 0.087 |
| 2014-2024 | 5.06 (-7.83 to 20.84) | 0.079 |
| **Middle Aged Adults (45-64)** | | |
| 1999-2017 | -2.63* (-2.84 to -2.44) | 0.002 |
| 2017-2021 | 3.46* (1.81 to 5.82) | 0.009 |
| 2021-2024 | -2.55* (-5.92 to -0.56) | 0.014 |
| **Older Adults (65+)** | | |
| 1999-2006 | -2.42* (-3.19 to -1.65) | 0.001 |
| 2006-2015 | -3.81* (-5.04 to -1.93) | <0.001 |
| 2015-2018 | -2.09* (-3.88 to -1.25) | 0.006 |
| 2018-2021 | 5.37* (3.56 to 6.59) | <0.001 |
| 2021-2024 | -1.85* (-3.33 to -0.83) | 0.0008 |
| APC = annual percent change; NH = non-Hispanic; * Indicates that the annual percentage change (APC) is significantly different from zero at α = 0.05. AAMR = age-adjusted mortality rate. The data for urbanization is only available till 2020 in the CDC Wonder Database. | | |

**Supplemental Table 4.** Overall and sex-stratified coronary artery disease–related age-adjusted mortality rates per 100,000, calculated using MCOD, in adults with cancer in the United States, 1999–2024.

| **Supplemental Table 4. Overall and sex-stratified coronary artery disease–related age-adjusted mortality rates per 100,000, calculated using MCOD, in adults with cancer in the United States, 1999–2024.** | | | |
| --- | --- | --- | --- |
| **Age-Adjusted Rate (95% CI)** | | | |
| **Year** | **Men** | **Women** | **Overall** |
| **1999** | 49.83 (49.29 - 50.38) | 19.99 (19.72 - 20.25) | 31.38 (31.12 - 31.64) |
| **2000** | 48.76 (48.22 - 49.29) | 19.55 (19.29 - 19.81) | 30.74 (30.48 - 30.99) |
| **2001** | 47.36 (46.84 - 47.88) | 18.90 (18.64 - 19.15) | 29.86 (29.61 - 30.12) |
| **2002** | 46.91 (46.40 - 47.43) | 18.61 (18.35 - 18.86) | 29.55 (29.30 - 29.80) |
| **2003** | 45.45 (44.96 - 45.95) | 17.88 (17.63 - 18.13) | 28.58 (28.34 - 28.83) |
| **2004** | 43.70 (43.22 - 44.19) | 17.11 (16.87 - 17.35) | 27.52 (27.28 - 27.75) |
| **2005** | 43.56 (43.08 - 44.04) | 17.10 (16.87 - 17.34) | 27.48 (27.25 - 27.72) |
| **2006** | 41.73 (41.27 - 42.19) | 16.24 (16.01 - 16.47) | 26.31 (26.08 - 26.54) |
| **2007** | 40.18 (39.73 - 40.63) | 15.60 (15.37 - 15.82) | 25.40 (25.18 - 25.62) |
| **2008** | 38.76 (38.33 - 39.19) | 14.87 (14.65 - 15.09) | 24.40 (24.19 - 24.62) |
| **2009** | 37.46 (37.04 - 37.88) | 14.14 (13.93 - 14.35) | 23.49 (23.28 - 23.70) |
| **2010** | 37.00 (36.59 - 37.42) | 13.92 (13.71 - 14.13) | 23.24 (23.04 - 23.45) |
| **2011** | 35.29 (34.90 - 35.69) | 13.26 (13.06 - 13.46) | 22.23 (22.03 - 22.43) |
| **2012** | 33.99 (33.61 - 34.38) | 12.55 (12.35 - 12.74) | 21.29 (21.09 - 21.48) |
| **2013** | 32.17 (31.80 - 32.53) | 11.97 (11.79 - 12.16) | 20.28 (20.09 - 20.46) |
| **2014** | 31.03 (30.68 - 31.39) | 11.15 (10.97 - 11.33) | 19.36 (19.18 - 19.54) |
| **2015** | 29.85 (29.51 - 30.20) | 10.72 (10.54 - 10.89) | 18.68 (18.50 - 18.85) |
| **2016** | 29.40 (29.07 - 29.74) | 10.56 (10.39 - 10.73) | 18.42 (18.25 - 18.59) |
| **2017** | 29.05 (28.72 - 29.38) | 10.20 (10.04 - 10.37) | 18.10 (17.93 - 18.27) |
| **2018** | 28.81 (28.49 - 29.13) | 10.40 (10.23 - 10.56) | 18.13 (17.97 - 18.30) |
| **2019** | 29.04 (28.72 - 29.36) | 10.20 (10.04 - 10.37) | 18.14 (17.98 - 18.30) |
| **2020** | 31.52 (31.19 - 31.85) | 11.30 (11.13 - 11.47) | 19.82 (19.65 - 19.99) |
| **2021** | 33.11 (32.77 - 33.46) | 12.03 (11.85 - 12.20) | 20.96 (20.78 - 21.14) |
| **2022** | 32.25 (31.92 - 32.58) | 11.63 (11.46 - 11.80) | 20.29 (20.12 - 20.46) |
| **2023** | 30.90 (30.58 - 31.22) | 11.16 (10.99 - 11.32) | 19.58 (19.41 - 19.74) |
| **2024** | 31.36 (31.04 - 31.68) | 11.21 (11.05 - 11.38) | 19.77 (19.61 - 19.94) |
| **Total** | **36.86 (36.46 - 37.27)** | **13.93 (13.72 - 14.13)** | **23.19 (22.99 - 23.39)** |

**Supplemental Table 5.** Race-stratified coronary artery disease–related age-adjusted mortality rates per 100,000, determined from MCOD, in adults with cancer in the United States, 1999–2024.

| **Supplemental Table 5. Race-stratified coronary artery disease–related age-adjusted mortality rates per 100,000, determined from MCOD, in adults with cancer in the United States, 1999–2024.** | | | | | |
| --- | --- | --- | --- | --- | --- |
| **Age-Adjusted Rate (95% CI)** | | | | | |
| **Year** | **NH American Indian or Alaska Native** | **NH Asian or Pacific Islander** | **NH Black** | **Hispanic** | **NH White** |
| **1999** | 17.23 (14.03 - 20.43) | 16.45 (15.09 - 17.82) | 31.74 (30.81 - 32.66) | 19.27 (18.26 - 20.28) | 31.77 (31.49 - 32.05) |
| **2000** | 17.65 (14.69 - 20.62) | 16.65 (15.31 - 17.98) | 30.91 (30.00 - 31.81) | 18.39 (17.43 - 19.35) | 31.11 (30.84 - 31.38) |
| **2001** | 15.85 (13.07 - 18.63) | 16.00 (14.74 - 17.25) | 30.80 (29.91 - 31.70) | 18.88 (17.94 - 19.82) | 30.19 (29.92 - 30.46) |
| **2002** | 17.85 (14.92 - 20.77) | 15.36 (14.19 - 16.53) | 30.10 (29.22 - 30.99) | 18.33 (17.43 - 19.24) | 29.91 (29.64 - 30.18) |
| **2003** | 20.11 (17.06 - 23.17) | 15.06 (13.93 - 16.19) | 28.67 (27.82 - 29.52) | 17.93 (17.06 - 18.81) | 28.97 (28.71 - 29.23) |
| **2004** | 18.52 (15.67 - 21.36) | 14.22 (13.16 - 15.29) | 27.13 (26.31 - 27.95) | 16.75 (15.94 - 17.57) | 27.98 (27.72 - 28.23) |
| **2005** | 14.42 (11.97 - 16.87) | 14.31 (13.29 - 15.34) | 27.14 (26.33 - 27.95) | 17.36 (16.55 - 18.17) | 28.88 (27.74 - 28.25) |
| **2006** | 15.65 (13.20 - 18.11) | 13.61 (12.64 - 14.57) | 25.18 (24.41 - 25.96) | 16.61 (15.83 - 17.38) | 26.85 (26.61 - 27.10) |
| **2007** | 16.95 (14.38 - 19.53) | 12.49 (11.58 - 13.39) | 24.87 (24.11 - 25.63) | 14.91 (14.20 - 15.63) | 25.94 (25.70 - 26.17) |
| **2008** | 16.52 (14.09 - 18.95) | 12.40 (11.52 - 13.27) | 23.65 (22.92 - 24.38) | 14.29 (13.61 - 14.96) | 24.95 (24.72 - 25.18) |
| **2009** | 14.71 (12.48 - 16.95) | 13.08 (12.21 - 13.95) | 22.09 (21.40 - 22.79) | 13.65 (13.01 - 14.30) | 24.04 (23.81 - 24.27) |
| **2010** | 18.44 (15.95 - 20.92) | 11.95 (11.13 - 12.76) | 21.90 (21.21 - 22.59) | 13.88 (13.24 - 14.52) | 23.82 (23.60 - 24.04) |
| **2011** | 16.29 (14.07 - 18.51) | 10.42 (9.70 - 11.15) | 21.39 (20.72 - 22.05) | 13.48 (12.88 - 14.09) | 22.79 (22.57 - 23.10) |
| **2012** | 15.86 (13.72 - 18.00) | 10.75 (10.03 - 11.47) | 19.77 (19.14 - 20.40) | 12.84 (12.27 - 13.41) | 21.90 (21.69 - 22.11) |
| **2013** | 13.65 (11.71 - 15.59) | 9.84 (9.18 - 10.50) | 18.57 (17.97 - 19.16) | 12.40 (11.85 - 12.94) | 20.97 (20.77 - 21.18) |
| **2014** | 12.98 (11.16 - 14.79) | 9.35 (8.73 - 9.97) | 17.67 (17.10 - 18.24) | 11.83 (11.32 - 12.35) | 20.05 (19.86 - 20.25) |
| **2015** | 14.46 (12.15 - 15.85) | 8.79 (8.21 - 9.36) | 17.11 (16.56 - 17.66) | 11.59 (11.10 - 12.09) | 19.37 (19.18 - 19.56) |
| **2016** | 12.91 (11.18 - 14.64) | 8.30 (7.76 - 8.85) | 16.79 (16.25 - 17.33) | 11.23 (10.76 - 11.71) | 19.12 (18.93 - 19.31) |
| **2017** | 13.63 (11.92 - 15.34) | 8.30 (7.77 - 8.82) | 16.20 (15.68 - 16.71) | 10.56 (10.12 - 11.01) | 18.83 (18.65 - 19.02) |
| **2018** | 12.73 (11.12 - 14.33) | 8.14 (7.63 - 8.65) | 16.18 (15.67 - 16.69) | 10.42 (9.99 - 10.86) | 18.92 (18.74 - 19.11) |
| **2019** | 13.01 (11.44 - 14.58) | 8.26 (7.76 - 8.76) | 15.90 (15.41 - 16.40) | 11.15 (10.70 - 11.59) | 18.97 (18.78 - 19.15) |
| **2020** | 15.55 (13.86 - 17.24) | 9.26 (8.75 - 9.77) | 18.33 (17.80 - 18.85) | 12.22 (11.77 - 12.67) | 20.62 (20.43 - 20.81) |
| **2021** | 14.01 (12.35 - 15.66) | 8.76 (8.25 - 9.28) | 18.73 (18.19 - 19.27) | 11.92 (11.48 - 12.37) | 22.09 (21.89 - 22.29) |
| **2022** | 12.19 (10.73 - 13.66) | 9.08 (8.57 - 9.58) | 18.55 (18.02 - 19.07) | 11.64 (11.22 - 12.07) | 21.31 (21.12 - 21.50) |
| **2023** | 13.24 (11.74 - 14.74) | 7.87 (7.42 - 8.33) | 17.50 (17.00 - 18.00) | 11.11 (10.70 - 11.52) | 20.69 (20.50 - 20.87) |
| **2024** | 13.36 (11.84 - 14.88) | 8.77 (8.28 - 9.25) | 17.94 (17.43 - 18.45) | 11.51 (11.10 - 11.93) | 20.86 (20.67 - 21.04) |
| **Total** | **15.28 (13.09 - 17.46)** | **11.44 (10.64 - 12.23)** | **22.10 (21.43 - 22.77)** | **14.00 (13.37 - 14.64)** | **23.84 (23.62 - 24.06)** |
| NH = Non-Hispanic | | | | | |

**Supplemental Table 6.** Age-stratified coronary artery disease–related age-adjusted mortality rates per 100,000, based on MCOD, in adults with cancer in the United States, 1999–2024.

| **Supplemental Table 6. Age-stratified coronary artery disease–related age-adjusted mortality rates per 100,000, based on MCOD, in adults with cancer in the United States, 1999–2024.** | | | |
| --- | --- | --- | --- |
| **Age-Adjusted Rate (95% CI)** | | | |
| **Year** | **Younger Adults (25-44)** | **Middle-Aged Adults (45-64)** | **Older Adults (65+)** |
| **1999** | 0.16 (0.14 - 0.19) | 8.14 (7.91 - 8.37) | 145.86 (144.58 - 147.13) |
| **2000** | 0.22 (0.19 - 0.25) | 8.10 (7.88 - 8.32) | 142.51 (141.26 - 143.77) |
| **2001** | 0.22 (0.18 - 0.25) | 7.96 (7.74 - 8.18) | 138.30 (137.07 - 139.52) |
| **2002** | 0.21 (0.18 - 0.24) | 7.65 (7.44 - 7.86) | 137.28 (136.07 - 138.50) |
| **2003** | 0.26 (0.23 - 0.30) | 7.53 (7.33 - 7.73) | 132.40 (131.21 - 133.59) |
| **2004** | 0.26 (0.23 - 0.30) | 7.37 (7.17 - 7.57) | 127.22 (126.06 - 128.37) |
| **2005** | 0.21 (0.18 - 0.24) | 7.35 (7.16 - 7.55) | 127.19 (126.05 - 128.34) |
| **2006** | 0.16 (0.14 - 0.19) | 6.98 (6.79 - 7.16) | 121.98 (120.86 - 123.09) |
| **2007** | 0.21 (0.18 - 0.24) | 6.76 (6.58 - 6.94) | 117.60 (116.51 - 118.68) |
| **2008** | 0.16 (0.14 - 0.19) | 6.53 (6.35 - 6.70) | 113.00 (111.94 - 114.05) |
| **2009** | 0.16 (0.14 - 0.19) | 6.31 (6.14 - 6.47) | 108.72 (107.70 - 109.75) |
| **2010** | 0.15 (0.13 - 0.18) | 6.21 (6.04 - 6.37) | 107.66 (106.64 - 108.67) |
| **2011** | 0.21 (0.17 - 0.24) | 6.15 (5.99 - 6.31) | 102.43 (101.46 - 103.41) |
| **2012** | 0.21 (0.18 - 0.24) | 5.75 (5.60 - 5.91) | 98.31 (97.36 - 99.26) |
| **2013** | 0.16 (0.13 - 0.19) | 5.64 (5.48 - 5.79) | 93.46 (92.55 - 94.37) |
| **2014** | 0.11 (0.09 - 0.13) | 5.40 (5.25 - 5.55) | 89.33 (88.45 - 90.21) |
| **2015** | 0.16 (0.13 - 0.19) | 5.38 (5.23 - 5.53) | 85.73 (84.88 - 86.58) |
| **2016** | 0.16 (0.14 - 0.19) | 5.36 (5.21 - 5.50) | 84.45 (83.61 - 85.29) |
| **2017** | 0.16 (0.13 - 0.19) | 5.36 (5.21 - 5.50) | 82.82 (82.01 - 83.64) |
| **2018** | 0.16 (0.13 - 0.20) | 5.34 (5.19 - 5.49) | 83.00 (82.20 - 83.81) |
| **2019** | 0.16 (0.13 - 0.19) | 5.30 (5.15 - 5.44) | 83.13 (82.34 - 83.92) |
| **2020** | 0.16 (0.13 - 0.19) | 5.91 (5.76 - 6.07) | 90.64 (89.82 - 91.46) |
| **2021** | 0.21 (0.18 - 0.24) | 5.85 (5.70 - 6.00) | 96.46 (95.60 - 97.32) |
| **2022** | 0.26 (0.23 - 0.30) | 5.85 (5.70 - 6.00) | 92.90 (92.08 - 93.71) |
| **2023** | 0.21 (0.18 - 0.24) | 5.51 (5.37 - 5.66) | 89.98 (89.18 - 90.78) |
| **2024** | 0.21 (0.18 - 0.24) | 5.50 (5.35 - 5.65) | 91.02 (90.21 - 91.82) |
| **Total** | **0.18 (0.16 - 0.22)** | **6.35 (6.18 - 6.52)** | **107.05 (106.06 - 108.04)** |

**Supplemental Table 7.** Coronary artery disease–related age-adjusted mortality rates per 100,000, stratified by census region, using MCOD, in adults with cancer in the United States, 1999–2024.

| **Supplemental Table 7. Coronary artery disease–related age-adjusted mortality rates per 100,000, stratified by census region, using MCOD, in adults with cancer in the United States, 1999–2024.** | | | | |
| --- | --- | --- | --- | --- |
|  | **Census Region: Northeast** | **Census Region: Midwest** | **Census Region:**  **South** | **Census Region:**  **West** |
| **Year** | **Age-Adjusted Rate (95% CI)** | **Age-Adjusted Rate (95% CI)** | **Age-Adjusted Rate (95% CI)** | **Age-Adjusted Rate (95% CI)** |
| **1999** | 37.25 (36.63 - 37.87) | 33.87 (33.32 - 34.43) | 27.26 (26.84 - 27.67) | 29.44 (28.87 - 30.01) |
| **2000** | 36.32 (35.71 - 36.93) | 32.63 (32.09 - 33.17) | 27.28 (26.87 - 27.69) | 28.45 (27.89 - 29.00) |
| **2001** | 34.79 (34.20 - 35.38) | 31.96 (31.42 - 32.49) | 26.55 (26.15 - 26.95) | 27.84 (27.30 - 28.39) |
| **2002** | 34.30 (33.72 - 34.89) | 31.59 (31.06 - 32.11) | 26.54 (26.14 - 26.94) | 27.24 (26.71 - 27.78) |
| **2003** | 32.79 (32.23 - 33.36) | 30.93 (30.41 - 31.45) | 25.59 (25.20 - 25.98) | 26.50 (25.98 - 27.02) |
| **2004** | 31.77 (31.22 - 32.33) | 29.94 (29.43 - 30.45) | 24.57 (24.20 - 24.95) | 25.31 (24.80 - 25.81) |
| **2005** | 30.84 (30.29 - 31.38) | 30.19 (29.68 - 30.70) | 24.73 (24.35 - 25.10) | 25.63 (25.13 - 26.13) |
| **2006** | 29.31 (28.78 - 29.84) | 29.38 (28.88 - 29.88) | 23.71 (23.34 - 24.07) | 24.28 (23.80 - 24.76) |
| **2007** | 28.72 (28.20 - 29.24) | 28.25 (27.76 - 28.73) | 22.92 (22.57 - 23.28) | 23.04 (22.57 - 23.50) |
| **2008** | 27.15 (26.64 - 27.65) | 27.46 (26.99 - 27.94) | 22.11 (21.76 - 22.45) | 22.16 (21.71 - 22.61) |
| **2009** | 25.46 (24.98 - 25.95) | 25.67 (25.21 - 26.12) | 21.88 (21.55 - 22.22) | 21.95 (21.51 - 22.39) |
| **2010** | 25.85 (25.36 - 26.34) | 25.02 (24.57 - 25.47) | 21.73 (21.40 - 22.06) | 21.21 (20.78 - 21.64) |
| **2011** | 24.37 (23.90 - 24.84) | 24.87 (24.43 - 25.32) | 20.40 (20.08 - 20.72) | 20.35 (19.94 - 20.77) |
| **2012** | 23.27 (22.81 - 23.73) | 23.41 (22.98 - 23.84) | 19.86 (19.55 - 20.16) | 19.50 (19.10 - 19.90) |
| **2013** | 21.45 (21.02 - 21.89) | 21.95 (21.54 - 22.36) | 19.40 (19.10 - 19.70) | 18.92 (18.53 - 19.31) |
| **2014** | 20.85 (20.42 - 21.27) | 21.54 (21.14 - 21.94) | 18.01 (17.73 - 18.30) | 18.12 (17.74 - 18.49) |
| **2015** | 19.76 (19.35 - 20.17) | 20.49 (20.10 - 20.88) | 17.86 (17.58 - 18.14) | 17.14 (16.79 - 17.50) |
| **2016** | 18.95 (18.54 - 19.35) | 20.06 (19.68 - 20.44) | 18.01 (17.73 - 18.29) | 17.04 (16.69 - 17.40) |
| **2017** | 18.04 (17.66 - 18.43) | 19.62 (19.24 - 20.10) | 18.03 (17.76 - 18.31) | 16.69 (16.34 - 17.03) |
| **2018** | 17.81 (17.43 - 18.19) | 19.50 (19.13 - 19.87) | 18.37 (18.10 - 18.64) | 16.61 (16.27 - 16.95) |
| **2019** | 17.34 (16.97 - 17.71) | 19.54 (19.17 - 19.91) | 18.66 (18.39 - 18.93) | 16.60 (16.27 - 16.94) |
| **2020** | 18.88 (18.50 - 19.27) | 21.56 (21.17 - 21.94) | 20.31 (20.03 - 20.59) | 18.04 (17.70 - 18.38) |
| **2021** | 18.76 (18.37 - 19.15) | 22.79 (22.39 - 23.19) | 22.07 (21.77 - 22.36) | 19.18 (18.82 - 19.54) |
| **2022** | 18.15 (17.78 - 18.52) | 21.49 (21.12 - 21.87) | 21.68 (21.40 - 21.96) | 18.52 (18.18 - 18.86) |
| **2023** | 17.46 (17.10 - 17.83) | 20.69 (20.32 - 21.06) | 21.37 (21.09 - 21.66) | 17.23 (16.91 - 17.56) |
| **2024** | 17.40 (17.04 - 17.76) | 21.26 (20.89 - 21.64) | 21.35 (21.07 - 21.63) | 17.68 (17.35 - 18.01) |
| **Total** | **24.88 (24.41 - 25.35)** | **25.21 (24.77 - 25.66)** | **21.93 (21.60 - 22.25)** | **21.33 (20.91 - 21.75)** |

**Supplemental Table 8.** Coronary artery disease–related age-adjusted mortality rates per 100,000, stratified by urban-rural classification, derived from MCOD, in adults with cancer in the United States, 1999–2020.

| **Supplemental Table 8. Coronary artery disease–related age-adjusted mortality rates per 100,000, stratified by urban-rural classification, derived from MCOD, in adults with cancer in the United States, 1999–2020.** | | |
| --- | --- | --- |
| **Age-Adjusted Rate (95% CI)** | | |
| **Year** | **Urban** | **Rural** |
| **1999** | 31.02 (30.73 - 31.31) | 33.04 (32.43 - 33.66) |
| **2000** | 30.20 (29.92 - 30.49) | 33.14 (32.53 - 33.75) |
| **2001** | 29.35 (29.08 - 29.63) | 32.01 (31.41 - 32.61) |
| **2002** | 28.88 (28.61 - 29.16) | 32.43 (31.83 - 33.03) |
| **2003** | 27.78 (27.51 - 28.04) | 32.11 (31.52 - 32.70) |
| **2004** | 26.65 (26.39 - 26.91) | 31.28 (30.70 - 31.87) |
| **2005** | 26.72 (26.46 - 26.98) | 31.04 (30.46 - 31.62) |
| **2006** | 25.44 (25.19 - 25.69) | 30.23 (29.67 - 30.80) |
| **2007** | 24.57 (24.33 - 24.81) | 29.15 (28.59 - 29.70) |
| **2008** | 23.48 (23.25 - 23.71) | 28.64 (28.09 - 29.19) |
| **2009** | 22.60 (22.37 - 22.82) | 27.73 (27.19 - 28.26) |
| **2010** | 22.32 (22.10 - 22.55) | 27.37 (26.84 - 27.89) |
| **2011** | 21.40 (21.19 - 21.62) | 25.93 (25.42 - 26.44) |
| **2012** | 20.49 (20.28 - 20.70) | 25.09 (24.59 - 25.59) |
| **2013** | 19.48 (19.28 - 19.68) | 24.11 (23.63 - 24.60) |
| **2014** | 18.53 (18.34 - 18.73) | 23.43 (22.95 - 23.90) |
| **2015** | 17.77 (17.59 - 17.96) | 22.98 (22.51 - 23.44) |
| **2016** | 17.52 (17.34 - 17.71) | 22.97 (22.51 - 23.44) |
| **2017** | 17.18 (17.17 - 17.36) | 22.65 (22.20 - 23.11) |
| **2018** | 17.19 (17.01 - 17.36) | 22.82 (22.36 - 23.27) |
| **2019** | 17.15 (16.98 - 17.32) | 23.20 (22.75 - 23.65) |
| **2020** | 18.70 (18.52 - 18.88) | 25.55 (25.08 - 26.03) |
| **Total** | **22.92 (22.70 - 23.15)** | **27.58 (27.05 - 28.11)** |
| The data for urbanization is only available till 2020 in the CDC Wonder Database. | | |
|  |  |  |

**Supplemental Table 9.** Coronary artery disease–related age-adjusted mortality rates per 100,000, stratified by U.S. states, determined using MCOD, in adults with cancer in the United States, 1999–2020.

| **Supplemental Table 9. Coronary artery disease–related age-adjusted mortality rates per 100,000, stratified by U.S. states, determined using MCOD, in adults with cancer in the United States, 1999–2020.** | |
| --- | --- |
| **State** | **Age-Adjusted Rate (95% CI)** |
| Alabama | 17.52 (17.22 - 17.82) |
| Alaska | 18.06 (16.93 - 19.18) |
| Arizona | 14.01 (13.77 - 14.24) |
| Arkansas | 22.49 (22.07 - 22.92) |
| California | 24.29 (24.15 - 24.42) |
| Colorado | 19.26 (18.91 - 19.60) |
| Connecticut | 22.84 (22.46 - 23.22) |
| Delaware | 25.25 (24.44 - 26.07) |
| District of Columbia | 23.14 (22.10 - 24.17) |
| Florida | 18.59 (18.46 - 18.73) |
| Georgia | 14.12 (13.91 - 14.34) |
| Hawaii | 16.43 (15.91 - 16.95) |
| Idaho | 19.35 (18.77 - 19.93) |
| Illinois | 21.62 (21.42 - 21.83) |
| Indiana | 25.16 (24.84 - 25.47) |
| Iowa | 26.13 (25.70 - 26.56) |
| Kansas | 19.86 (19.45 - 20.27) |
| Kentucky | 27.69 (27.29 - 28.10) |
| Louisiana | 16.26 (15.95 - 16.57) |
| Maine | 24.91 (24.29 - 25.54) |
| Maryland | 27.50 (27.14 - 27.86) |
| Massachusetts | 19.70 (19.44 - 19.97) |
| Michigan | 25.48 (25.23 - 25.73) |
| Minnesota | 25.74 (25.39 - 26.09) |
| Mississippi | 23.78 (23.32 - 24.24) |
| Missouri | 23.55 (23.25 - 23.86) |
| Montana | 17.27 (16.64 - 17.89) |
| Nebraska | 28.29 (27.68 - 28.89) |
| Nevada | 12.39 (12.02 - 12.76) |
| New Hampshire | 24.46 (23.79 - 25.13) |
| New Jersey | 25.71 (25.44 - 25.97) |
| New Mexico | 17.19 (16.72 - 17.66) |
| New York | 25.43 (25.26 - 25.61) |
| North Carolina | 21.98 (21.74 - 22.23) |
| North Dakota | 30.77 (29.78 - 31.77) |
| Ohio | 31.98 (31.73 - 32.24) |
| Oklahoma | 29.46 (29.02 - 29.90) |
| Oregon | 22.90 (22.52 - 23.27) |
| Pennsylvania | 28.42 (28.20 - 28.63) |
| Rhode Island | 34.20 (33.37 - 35.04) |
| South Carolina | 19.73 (19.40 - 20.06) |
| South Dakota | 26.93 (26.08 - 27.79) |
| Tennessee | 25.83 (25.51 - 26.15) |
| Texas | 23.67 (23.49 - 23.84) |
| Utah | 11.77 (11.37 - 12.17) |
| Vermont | 33.84 (32.73 - 34.95) |
| Virginia | 17.52 (17.27 - 17.76) |
| Washington | 22.61 (22.31 - 22.91) |
| West Virginia | 35.86 (35.22 - 36.50) |
| Wisconsin | 21.98 (21.68 - 22.29) |
| Wyoming | 20.17 (19.19 - 21.15) |

**Supplemental Table 10.** Cancer and coronary artery disease–related age-adjusted mortality rates per 100,000, stratified by cancer subtype, based on MCOD, in adults in the United States, 1999–2024.

| **Supplemental Table 10. Cancer and coronary artery disease–related age-adjusted mortality rates per 100,000, stratified by cancer subtype, based on MCOD, in adults in the United States, 1999–2024.** | | | | | |
| --- | --- | --- | --- | --- | --- |
| **Age-Adjusted Rate (95% CI)** | | | | | |
| **Year** | **Lung Cancer** | **Gastrointestinal Cancer** | **Breast Cancer** | **Prostate Cancer** | **Hematological Cancer** |
| **1999** | 6.61 (6.49 - 6.73) | 6.74 (6.62 - 6.86) | 2.48 (2.40 - 2.55) | 4.52 (4.42 - 4.62) | 3.47 (3.38 - 3.55) |
| **2000** | 6.57 (6.45 - 6.69) | 6.69 (6.57 - 6.81) | 2.37 (2.30 - 2.44) | 4.30 (4.21 - 4.40) | 3.56 (3.47 - 3.65) |
| **2001** | 6.54 (6.42 - 6.65) | 6.36 (6.24 - 6.47) | 2.32 (2.25 - 2.40) | 4.27 (4.17 - 4.36) | 3.33 (3.24 - 3.41) |
| **2002** | 6.52 (6.40 - 6.63) | 6.29 (6.18 - 6.41) | 2.24 (2.17 - 2.31) | 4.17 (4.08 - 4.27) | 3.36 (3.27 - 3.44) |
| **2003** | 6.40 (6.29 - 6.52) | 5.96 (5.85 - 6.07) | 2.07 (2.00 - 2.13) | 4.00 (3.91 - 4.09) | 3.32 (3.23 - 3.40) |
| **2004** | 6.20 (6.09 - 6.31) | 5.66 (5.56 - 5.77) | 1.99 (1.93 - 2.05) | 3.77 (3.69 - 3.86) | 3.22 (3.14 - 3.30) |
| **2005** | 6.27 (6.16 - 6.38) | 5.69 (5.58 - 5.79) | 1.93 (1.87 - 1.99) | 3.68 (3.59 - 3.76) | 3.25 (3.17 - 3.33) |
| **2006** | 6.07 (5.96 - 6.18) | 5.51 (5.40 - 5.61) | 1.88 (1.82 - 1.94) | 3.56 (3.47 - 3.64) | 3.00 (2.92 - 3.07) |
| **2007** | 5.86 (5.75 - 5.96) | 5.21 (5.11 - 5.31) | 1.78 (1.72 - 1.84) | 3.40 (3.32 - 3.48) | 3.01 (2.93 - 3.08) |
| **2008** | 5.63 (5.53 - 5.73) | 4.95 (4.85 - 5.05) | 1.68 (1.62 - 1.73) | 3.25 (3.17 - 3.33) | 2.91 (2.84 - 2.99) |
| **2009** | 5.48 (5.38 - 5.58) | 4.61 (4.51 - 4.70) | 1.60 (1.55 - 1.66) | 3.13 (3.06 - 3.21) | 2.80 (2.72 - 2.87) |
| **2010** | 5.52 (5.42 - 5.62) | 4.59 (4.50 - 4.68) | 1.61 (1.56 - 1.66) | 2.94 (2.86 - 3.01) | 2.84 (2.76 - 2.91) |
| **2011** | 5.22 (5.12 - 5.32) | 4.36 (4.27 - 4.45) | 1.57 (1.51 - 1.62) | 2.81 (2.74 - 2.88) | 2.72 (2.65 - 2.79) |
| **2012** | 4.99 (4.90 - 5.09) | 4.16 (4.08 - 4.25) | 1.45 (1.40 - 1.50) | 2.70 (2.64 - 2.77) | 2.59 (2.52 - 2.66) |
| **2013** | 4.69 (4.60 - 4.78) | 4.00 (3.92 - 4.08) | 1.30 (1.25 - 1.35) | 2.54 (2.48 - 2.61) | 2.51 (2.44 - 2.57) |
| **2014** | 4.39 (4.31 - 4.48) | 3.83 (3.75 - 3.91) | 1.22 (1.17 - 1.26) | 2.41 (2.34 - 2.47) | 2.45 (2.38 - 2.51) |
| **2015** | 4.18 (4.09 - 4.26) | 3.70 (3.63 - 3.78) | 1.20 (1.15 - 1.24) | 2.31 (2.25 - 2.38) | 2.37 (2.31 - 2.44) |
| **2016** | 4.08 (4.00 - 4.16) | 3.65 (3.58 - 3.73) | 1.17 (1.13 - 1.21) | 2.32 (2.26 - 2.38) | 2.36 (2.30 - 2.43) |
| **2017** | 4.00 (3.92 - 4.08) | 3.55 (3.47 - 3.62) | 1.13 (1.09 - 1.17) | 2.25 (2.19 - 2.31) | 2.32 (2.26 - 2.38) |
| **2018** | 3.92 (3.85 - 4.00) | 3.63 (3.56 - 3.71) | 1.15 (1.10 - 1.19) | 2.25 (2.19 - 2.31) | 2.31 (2.25 - 2.37) |
| **2019** | 3.87 (3.80 - 3.95) | 3.67 (3.60 - 3.75) | 1.12 (1.08 - 1.16) | 2.30 (2.25 - 2.36) | 2.35 (2.29 - 2.41) |
| **2020** | 4.04 (3.97 - 4.12) | 3.92 (3.85 - 4.00) | 1.28 (1.23 - 1.32) | 2.66 (2.60 - 2.72) | 2.65 (2.58 - 2.71) |
| **2021** | 4.22 (4.15 - 4.30) | 4.10 (4.03 - 4.18) | 1.33 (1.29 - 1.38) | 2.78 (2.71 - 2.84) | 2.86 (2.79 - 2.92) |
| **2022** | 4.03 (3.95 - 4.10) | 4.02 (3.94 - 4.09) | 1.31 (1.27 - 1.35) | 2.61 (2.54 - 2.67) | 2.71 (2.65 - 2.78) |
| **2023** | 3.90 (3.83 - 3.97) | 3.93 (3.85 - 4.00) | 1.23 (1.19 - 1.28) | 2.56 (2.50 - 2.62) | 2.51 (2.45 - 2.57) |
| **2024** | 3.83 (3.76 - 3.90) | 4.03 (3.95 - 4.10) | 1.21 (1.17 - 1.25) | 2.65 (2.59 - 2.72) | 2.54 (2.48 - 2.60) |
| **Total** | **5.11 (5.02 - 5.21)** | **4.72 (4.63 - 4.81)** | **1.60 (1.54 - 1.65)** | **3.08 (3.00 - 3.15)** | **2.82 (2.74 - 2.89)** |
